# Peptidomics of the Agriculturally Damaging Larval Stage of the Cabbage Root Fly *Delia radicum* (Diptera: Anthomyiidae)

**DOI:** 10.1371/journal.pone.0041543

**Published:** 2012-07-25

**Authors:** Judith Zoephel, Wencke Reiher, Karl-Heinz Rexer, Jörg Kahnt, Christian Wegener

**Affiliations:** 1 Department of Biology, Animal Physiology, Philipps-University Marburg, Marburg, Germany; 2 Department of Biology, Mycology, Philipps-University Marburg, Marburg, Germany; 3 Max-Planck-Institute of Terrestrial Microbiology, Marburg, Germany; 4 Neurobiology and Genetics, Theodor Boveri Institute, Biocenter, University of Würzburg, Würzburg, Germany; U. Kentucky, United States of America

## Abstract

The larvae of the cabbage root fly induce serious damage to cultivated crops of the family Brassicaceae. We here report the biochemical characterisation of neuropeptides from the central nervous system and neurohemal organs, as well as regulatory peptides from enteroendocrine midgut cells of the cabbage maggot. By LC-MALDI-TOF/TOF and chemical labelling with 4-sulfophenyl isothiocyanate, 38 peptides could be identified, representing major insect peptide families: allatostatin A, allatostatin C, FMRFamide-like peptides, kinin, CAPA peptides, pyrokinins, sNPF, myosuppressin, corazonin, SIFamide, sulfakinins, tachykinins, NPLP1-peptides, adipokinetic hormone and CCHamide 1. We also report a new peptide (Yamide) which appears to be homolog to an amidated eclosion hormone-associated peptide in several *Drosophila* species. Immunocytochemical characterisation of the distribution of several classes of peptide-immunoreactive neurons and enteroendocrine cells shows a very similar but not identical peptide distribution to *Drosophila*. Since peptides regulate many vital physiological and behavioural processes such as moulting or feeding, our data may initiate the pharmacological testing and development of new specific peptide-based protection methods against the cabbage root fly and its larva.

## Introduction

The cabbage root fly *Delia radicum* is a serious pest species on cultivated Brassicaceae (e.g. cabbage, turnip, swede) in the temperate holarctic region. Up to 60–90% of untreated brassica crops can be regionally damaged by a cabbage root fly infestation, while average losses of untreated crop may be somewhat above 20% (see [1], [2] for review). The damaging life stage of *D. radicum* is the larva also known as cabbage maggot. After hatching from eggs deposited at the root base close to the ground, larvae first feed on smaller rootlets. Later on, with growing size, they also attack the main root. This largely subterranean life of the larva and the long emergence period of the adult flies make pesticide control difficult and ineffective [3]. In recent time, *D. radicum* has enlarged its host range and is now attacking rapeseed (*Brassica napus* L.) in several countries, in Germany and Czech Republic since the mid-1990ies [3], [4]. Since rapeseed monocultures have increased considerably due to biofuel and oil production, *D. radicum* is causing considerable economic losses additional to the damage to food crops.

Neuropeptides and peptides from endocrine cells (together referred to as regulatory peptides) and especially their synthetic mimetics with improved bioavailability and peptidase-resistance have a high potential for specific and environmentally friendly pest control since they can regulate feeding, development and reproduction (see [5], [6]). Though the chemical or biotechnological synthesis of peptides is still comparatively expensive, bioactive peptides can potentially be ectopically expressed in transgenic plants. Also peptide uptake through the insect cuticle or gut can be considerably enhanced by lipophilic and degradation-resistant analogues (e.g. [7]), or by coupling to molecules like lectins which are transported through the gut epithelium [8].

Here we report the mass spectrometric characterisation of 38 peptides (including variants of different size and N-terminal pyroglutamination) from the central nervous system (CNS) and midgut of *D. radicum* larvae. We further describe the cellular distribution of selected sets of peptidergic neurons and enteroendocrine cells, and morphologically characterise the major neurohemal organs of this species. Genomic or EST data are not available for *D. radicum*, and at the onset of our work, no peptide sequence data were available for this species. Recently, however, Audsley and colleagues [9] elucidated the sequence of 20 neuropeptides including variants from the CNS of adult *D. radicum*. Our results confirm the occurrence of all but two of these neuropeptides also in the damaging life stage (i.e. the larva), and reveal further peptides and peptide families that are either absent or have so far not been found in adult flies. The now available peptide data may initiate the development of new specific peptide-based protection methods against the difficult-to-control cabbage root fly.

## Materials and Methods

### Insects

Adult *D. radicum* were reared at 20°C and an L:D cycle of 16∶8 in a small flight cage [10]. Both dry and wet food was provided. The dry food consisted of dextrose, skim milk powder, soy flour and brewer’s yeast in a 10∶10:1∶1 ratio. The wet food consisted of honey, soy flower and brewer’s yeast in a 5∶5:1 ratio, if necessary diluted with water. For egg deposition, small pieces of swedes were placed into the fly cage. Swede pieces with deposited eggs were then transferred to breeding boxes (Phytacon vessel, Carl Roth, Karlsruhe, Germany) filled with autoclaved bird sand to prevent mould. After approximately three weeks the first pupae appeared on the sand’s surface and were transferred to the fly cage again where adult flies eclosed after about one week.

*Drosophila virilis* were raised on standard *Drosophila* medium at 18°C or 25°C at L:D 12∶12.

### Peptide Extraction

Larval ring glands (RGs), central nervous systems (CNS) and midgut tissue were dissected on ice in HL3 saline (80 mM NaCl, 5 mM KCl, 1.5 mM CaCl_2_, 20 mM MgCl_2_, 10 mM NaHCO_3_, 5 mM trehalose, 115 mM sucrose, 5 mM HEPES, adjusted to pH of 7.2 with HCl; [11]) using fine forceps and scissors. The tissues were immediately transferred into 40–60 µl extraction solution (90% methanol, 9% gradient grade water, 1% trifluoroacetic acid (TFA) (v/v)) in an Eppendorf low bind tube and incubated for 30 min on ice. CNS were additionally sonicated in a water bath for 15 min to homogenize tissue before incubating 30 min on ice. Subsequently, the samples were centrifuged at 18,000 g for 15 min and the supernatant (peptides dissolved in extraction solution) was transferred to a fresh Eppendorf low bind tube. 10 µl HPLC grade water was added to the extract and methanol was removed by concentrating the sample to 10 µl in a vacuum centrifuge. The concentrated sample was stored at −20°C until further use.

### Peptide Coupling with 4-sulfophenyl Isothiocyanate (SPITC) for LC/MS

Based on the method described by Wang *et al.* [12], the concentrated samples were dissolved in 8 µl solvent (50% acetonitrile, 0.01% TFA, 49.99% HPLC grade water (v/v/v)) and sonicated for 20 min in an ultrasonic water bath. After that, the samples were centrifuged for 15 min at 18,000 g and the supernatant was transferred to a fresh Eppendorf low bind tube. 30 µg/µl SPITC (4-Isothio-cyanatobenzenesulfonic acid, Sigma-Aldrich) was added to yield a 92 mM SPITC solution. Then, 3 µl buffer (136 mM (NH_4_)_2_CO_3_) was added and after incubating for 30 min at 55°C, the sample was concentrated to a volume of 10 µl by vacuum centrifugation. Then, 20 µl of 0.5% acetic acid were added and the sample was subjected to HPLC.

### Capillary RP-HPLC

The concentrated unlabelled samples were dissolved in 40–60 µl eluent A (98% HPLC grade water, 2% acetonitrile, 0.05% TFA (v/v/v)) for 30 min at room temperature and sonicated for 20 min in a water bath. After centrifugation for 15 min at 18,000 g, the supernatant was transferred to a fresh low bind Eppendorf tube and injected into an UltiMate 3000 capillary HPLC system (Dionex, Idstein, Germany) connected to a Proteineer Fraction Collector (Bruker Daltonik GmbH, Bremen, Germany). SPITC-labelled samples were injected in 0.5% acetic acid. The samples were loaded onto a RP C18 trap column (Acclaim PepMap100 C18, 5 µm, 100 Å) with eluent A at a flow rate of 20 µl/min. Then the flow was switched through the trap column and the analytical RP column (Acclaim PepMap100 C18, 3 µm, 100 Å) with a rate of 2 µl/min. Peptides were eluted with a linear gradient from 4%−60% eluent B (80% acetonitrile, 20% HPLC grade water, 0.04% TFA (v/v/v)) in 30 min. 1 µl sample fraction mixed with 1 µl of matrix solution (half-saturated recrystallised α-cyano-4-hydroxycinnamic acid in 60% acetonitrile, 40% HPLC grade water, 0.1% TFA (v/v/v)) was spotted every 30 s onto a stainless steel MALDI target plate (Applied Biosystems/MDS SCIEX, Foster City, CA, USA).

### Sample Preparation for Direct MS Peptide Profiling

Direct peptide profiling was performed on single larval tissues and the dorsal sheath of the adult thoracico-abdominal ganglion (TAG) as described [13]. The tissues were dissected in saline (128 mM NaCl, 2 mM KCl, 1.8 mM CaCl_2_, 4 mM MgCl_2_, 36 mM sucrose, 5 mM HEPES, adjusted to pH 7.1 with NaOH; [14]), briefly rinsed in a fresh droplet of Aqua bidest, and then transferred onto a stainless steel MALDI target plate. A small amount of matrix solution (saturated recrystallised α-cyano-4-hydroxycinnamic acid in 30% methanol, 30% ethanol, 0.1% TFA (v/v)) was added with a manual oocyte injector (Drummond Digital, Broomall, PA, USA).

### MALDI TOF MS/MS

Masses were analysed with a 4800 *Plus* MALDI TOF/TOF Analyser (Applied Biosystems/MDS SCIEX, Foster City, CA, USA) at a laser wavelength of 355 nm. Settings like laser intensity and the number of sub-spectra per plate spot varied among the samples and were adjusted individually. The device was calibrated with a peptide calibration standard (Applied Biosystems Calibration Mixture 2). Peptides from the LC/MS samples were fragmented by post-source decay (PSD). For direct tissue profiling, both PSD and collision-induced dissociation (CID) were applied depending on sample condition. MS/MS spectra were interpreted using Data Explorer 4.10 software (Applied Biosystems/MDS SCIEX, Foster City, CA, USA).

### Data Base Entry

The peptide sequences have been submitted to the Uniprot database (http://www.uniprot.org/); accession numbers are listed in Table 1.

**Table 1:** Sequences, accession numbers and tissue distribution of the peptides characterised in *D. radicum* larvae.

Peptide	Sequence^a^	Mass [M+H]^+^	UniProt Accession	CNS	ring gland	tPSOs^b^	aPSOs^b^	midgut	SPITC-labeled^c^	detected inadults [9]
**A-type allatostatins**										
AST-A_909_	ARPYSFGLa	909.50	B3EWI2	Y				Y	Y	Y
AST-A_921_^d^	LPVYNFGLa	921.43	B3EWL8					Y		Y
AST-A_952_	NRPYSFGLa	952.49	B3EWJ3	Y				Y	Y	Y
AST-A_953_	VERYAFGLa	953.53	B3EWJ4	Y				Y	Y	Y
**C-type allatostatins**										
AST-C	pQVRYRQcYFNPIScF	1904.90	B3EWJ5	Y				Y		
AST-C	QVRYRQcYFNPIScF	1921.87	B3EWJ6	Y				Y		
**FMRFamide-like peptides**										
FMRFa_885_	GDNFMRFa	885.42	B3EWJ7	Y					Y	
FMRFa_899_	GQDFMRFa	899.42	B3EWJ8	Y					Y	
FMRFa_925_	PDNFMRFa	925.44	B3EWJ9	Y		Y			Y	Y
FMRFa_942_	GGNDFMRFa	942.44	B3EWK0	Y		Y			Y	Y
FMRFa_971_^d^	EQDFMRFa	971.50	-			Y				Y
FMRFa_996_	PGQDFMRFa	996.48	B3EWK1	Y		Y				
FMRFa_1067_	APGQDFMRFa	1067.51	B3EWK2	Y		Y				Y
FMRFa_1097_	TPGQDFMRFa	1097.60	B3EWK3	Y		Y			Y	Y
FMRFa_1154_	SAPGQDFMRFa	1154.54	B3EWK4	Y		Y				?^e^
FMRFa_1181_	LPEQDFMRFa	1181.60	B3EWK5	Y		Y			Y	?^e^
FMRFa_1185_	SAQGQDFMRFa	1185.53	B3EWK8	Y		Y			Y	
**Yamides**										
Ya	LPSIGHYYa	948.50	B3EWK9	Y	Y				Y	
**Kinins**										
Kinin	NSVVLGKKQRFHSWGa	1741.40	B3EWL0	Y						
**putative CAPA-peptides**										
CAPA-pyrokinin	AGPSATTGVWFGPRLa	1515.81	B3EWL1	Y	Y		Y		Y	
CAPA-pyrokinin^2–15^	GPSATTGVWFGPRLa	1444.78	B3EWL2	Y	Y				Y	
CAPA-periviscerokinin-1	GGGGTSGLFAFPRVa	1321.72	B3EWL3	Y			Y		Y	
CAPA-periviscerokinin-2	AGLFAQPRLa	971.59	B3EWL4	Y			Y		Y	
**putative HUGIN-peptides**										
HUG-pyrokinin	SVQFKPRLa	973.59	B3EWL5	Y	Y					Y
**short neuropeptide Fs**										
sNPF-1^4–11^	SPSLRLRFa	974.61	B3EWL6	Y	Y				Y	Y
sNPF-1	AQRSPSLRLRFa	1329.80	B3EWL7	Y	Y				Y	Y
**Myosuppressin**										
Myosuppressin	TDVDHVFLRFa	1247.70	B3EWL9	Y	Y				Y	Y
Myosuppressin^2–10^	DVDHVFLRFa	1146.59	B3EWM0	Y						
**Corazonin**										
Corazonin	pQTFQYSRGWTNa	1369.69	B3EWM1	Y	Y					Y
Corazonin^3–11^	FQYSRGWTNa	1157.56	B3EWM2	Y					Y	
**SIFamides**										
SIFa	AYRKPPFNGSIFa	1395.74	B3EWH1	Y					Y	
**Sulfakinins**										
Sulfakinin	GGEEQFDDYGHMRFa	1686.68	B3EWM3	Y					Y	Y
Sulfakinin^6–14^	FDDYGHMRFa	1186.52	B3EWM4	Y					Y	Y
**Tachykinin-related peptides**										
TK_1010_	TPTAFYGVRa	1010.55	B3EWM5	Y				Y	Y	
TK_1116_^f^	GLGNNAFLGVRa	1116.62	B3EWM6	Y				Y	Y	
**NPLP1-peptides**										
APK^g^	SVAALAAQGLL[YNAPK]	1586.85	B3EWM9	Y					Y	
**Adipokinetic hormones**										
AKH	pQLTFSPDWa	975.48	B3EWM7		Y					Y
AKHGK	pQLTFSPDWGK	1161.62			Y					Y
AKHGKR	pQLTFSPDWGKR	1317.65			Y					
**CCHamide 1 peptides**										
CCHa 1	ScLEYGHScWGAHa	1446.56	B3EWM8					Y		

a)Leu and Ile have the same molecular mass. Since we did not obtain distinguishing high-energy collision w-fragments [76], we are unable to distinguish between these two amino acids. Therefore, Leu and Ile in the sequences above have to be considered as predicted only based on the homolog peptides from *Drosophila* or other Dipterans. Small letter c within a sequence indicates cysteines that form an intramolecular disulfide bridge.

b)tPSO  =  thoracic PSO, aPSO  =  abdominal PSO, data from direct profiling of the dorsal sheath of the adult thoracico-abdominal ganglion.

c)these peptides could be sequenced in their SPITC-labelled form.

d)Mass peak indicative of this peptide appeared consistently, but could not be fragmented. Sequence adapted from [9].

e)a peptide with similar mass but different sequence (SPKQDFMRFa, 1154.6 Da and KPNQDFMRFa, 1181.6 Da) was reported by Audsley et al. [9].

f)The y9-fragment identifying the sequence order of positions 2–3 could not be found in SPITC-labelled and unlabeled spectra. The sequence LG is assumed since a very similar tachykinin (*Cav*-TKII: GLGNNAFVGVRa) was isolated and Edman-sequenced from the blowfly *Calliphora vomitoria* [73].

g)Only amino acids 1–11 of APK have been fully fragmented and are sequence identical to the N-terminus of APK of *Drosophila melanogaster* [24], [28]. The y-fragment representing amino acids 12–16 matches the mass of amino acids 12–15 of *Drosophila* APK plus the mass of tyrosine. Therefore, we assume the listed sequence. The position of the tyrosine and the C-terminal NAPK is not confirmed by fragmentation data.

### Immunostainings

CNS with and without RG attached were dissected on ice in HL3 saline and immediately fixed in 4% paraformaldehyde in 0.01 M phosphate-buffered saline (PBS), pH 7.1 for 3.5 h at 4°C. Afterwards tissues were washed 5 times for 10 min in PBT (0.1 M PBS with 0.3% TritonX) on a shaker at room temperature (RT). Preincubation with 10% normal goat serum (Dianova, Hamburg, Germany) in PBT for 4 h at RT on a shaker was followed by the incubation with primary antisera diluted in PBT and 10% normal goat serum for 2 days at RT on a shaker. The following polyclonal rabbit primary antibodies were used: anti-Dip-AST-A (kind gift of Hans Agricola, Jena, Germany [15]) diluted 1∶5000, anti-RFamide (kind gift of Eve Marder, Brandeis, USA [16]) diluted 1∶4000, anti-SIFa (kind gift of Peter Verleyen and Liliane Schoofs, Leuven, Belgium [17]) diluted 1∶500, anti-DH31 (kind gift of Jan Veenstra, Bordeaux, France [18]), anti-Lem-Tachykinin-related peptide (kind gift of Dick Nässel, Stockholm, Sweden [19]), anti-MIP and anti-PRXa (kind gift of Manfred Eckert, Jena, Germany [20], [21] diluted 1∶5000. The mouse monoclonal anti-PDF serum (donated by Justin Blau, obtained from the Developmental Studies Hybridoma Bank developed under the auspices of the NICHD and maintained by The University of Iowa, Department of Biology, Iowa City, USA) was diluted 1∶100.

After three washing steps with PBT, the samples were incubated with affinity-purified goat-anti rabbit or goat-anti mouse Cy3 or Cy5 IgG (Jackson Immunoresearch, Pa., USA) diluted 1∶100 in PBT and 10% normal goat serum for 2 days in constant darkness on a shaker. 3 washing steps of 10 min in PBT followed before a final wash in PBS. Tissues were mounted in 80% glycerol in 0.1 M PBS and analyzed with a confocal laser scanning microscope (TCS SP5, Leica, Wetzlar, Germany).

### Scanning Electron Microscopy

Samples were dissected in HL3 saline and fixed in 5% glutaraldehyde in 0.1 M PBS, pH 7.1, for 2 h at 4°C. Then, the samples were briefly dipped into chloroform and fixed further in 5% glutaraldehyde as above overnight. After washing, the samples were postfixed for 2 h in osmiumtetroxide (1% in 0.1 M Sörensen buffer, pH 7.2). Fixed samples were washed in Sörensen buffer and water, dehydrated in ethylene-glycol monoethylether over night followed by three 10 min changes in 100% acetone, and critical-point-dried using a Polaron E3000 (Balzer Union). Afterwards, samples were sputtered with gold particles with a sputter coater (Balzer Union), and then examined on a Hitachi S-530 scanning electron microscope.

## Results

### LC-MS/MS of Ring Gland Extracts

To characterise the sequence of *D. radicum* neuropeptides, we started with an LC-MS/MS analysis of extracts from 10–40 pooled ring glands (2 runs without, 4 runs with SPITC labelling). Automatic PSD peptide fragmentation was based first on a mass list containing the masses obtained by direct profiling (see below) and masses of biochemically identified *Drosophila* peptides, and subsequently on signal intensity. Some of the extracted peptide samples were coupled with 4-sulfophenyl isothiocyanate (SPITC) to direct fragmentation towards y-fragments [12], [22]. The selective enhancement of y-fragments after SPITC labelling strongly decreases the complexity of PSD fragmentation patterns. This facilitates the interpretation of fragment spectra in general [23], and also improved *de novo* sequencing of *D. radicum* peptides considerably.

**Figure 1:**
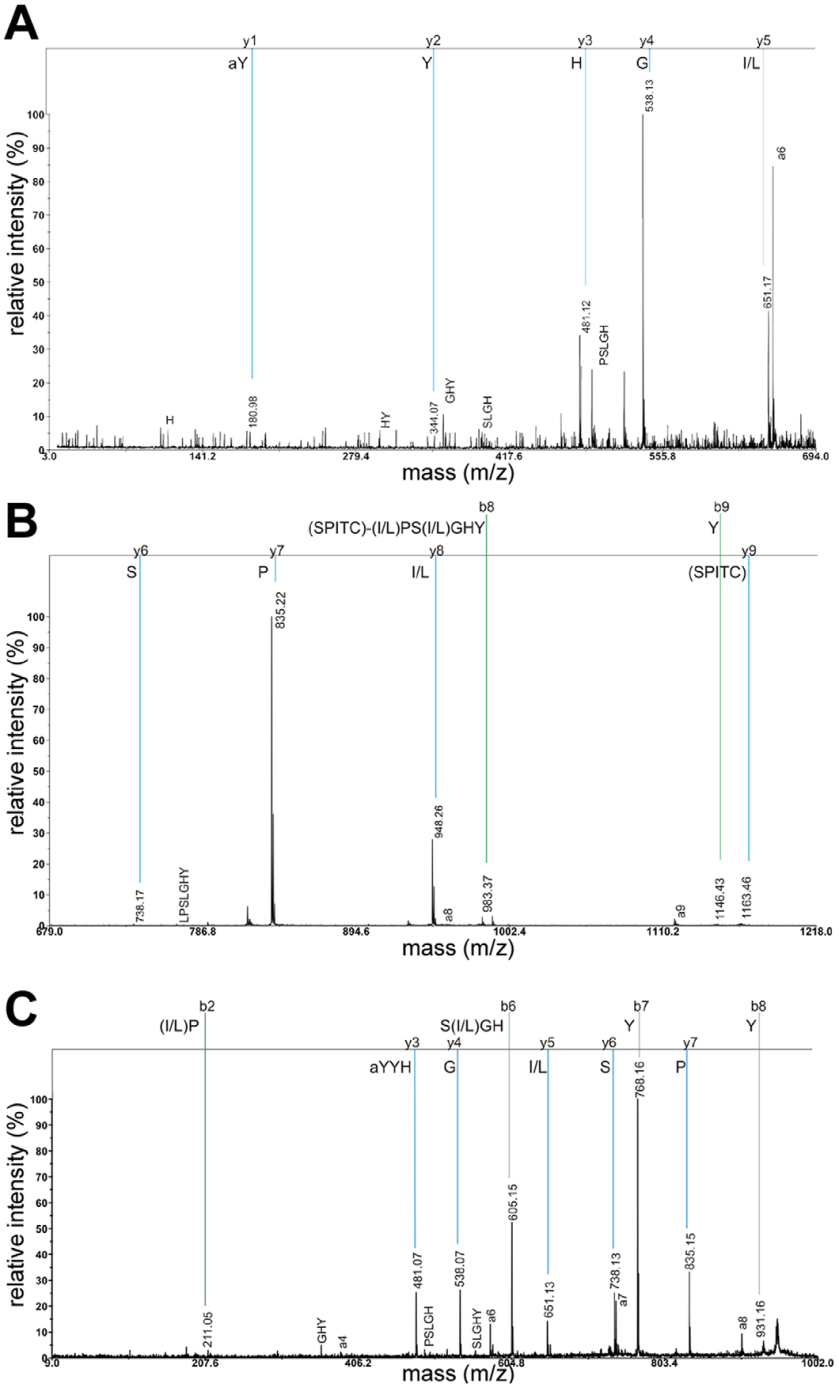
MS/MS spectrum of Yamide. A+B) SPITC-labelled; C) unlabeled. A+B) The fragment spectrum was divided, therefore the relative intensities vary. y-fragments are labelled with blue lines, b-fragments with green lines. Internal and a-fragments are shown as well.

The LC/MS-analysis revealed the presence of HUG-PK, sNPF-1, sNPF-1^4–11^, AKH, AKHGK (a processing intermediate of AKH), myosuppressin and corazonin in the ring gland. All peptide sequences were validated by fragmentation (see Table 1). Interestingly, we found and fragmented the [M+H]^+^ adduct of AKH (975.5 Da, Fig. S1), which in *Drosophila* and other insects is only found as a sodium or potassium adduct (e.g. [24], [25], [26]). SPITC labelling of an unknown peptide ion with the mass of 948.5 Da yielded a full y-fragment spectrum (Fig. 1). Since leucine and isoleucine are mass-identical and cannot be distinguished based on y-fragments, this fragment spectrum indicates the amino acid sequence (L/I)PS(L/I)GHYYamide. The C-terminal amidation is a unique modification of bioactive neuropeptides [27], hence the sequence and occurrence in the ring gland suggest that this peptide -designated here as Yamide- may be stored and released as a bioactive peptide hormone. Yamide shows no sequence-similarity with any hitherto sequenced insect peptide, suggesting it constitutes a new insect peptide family.

### Neuropeptides from the Central Nervous System

**Figure 2:**
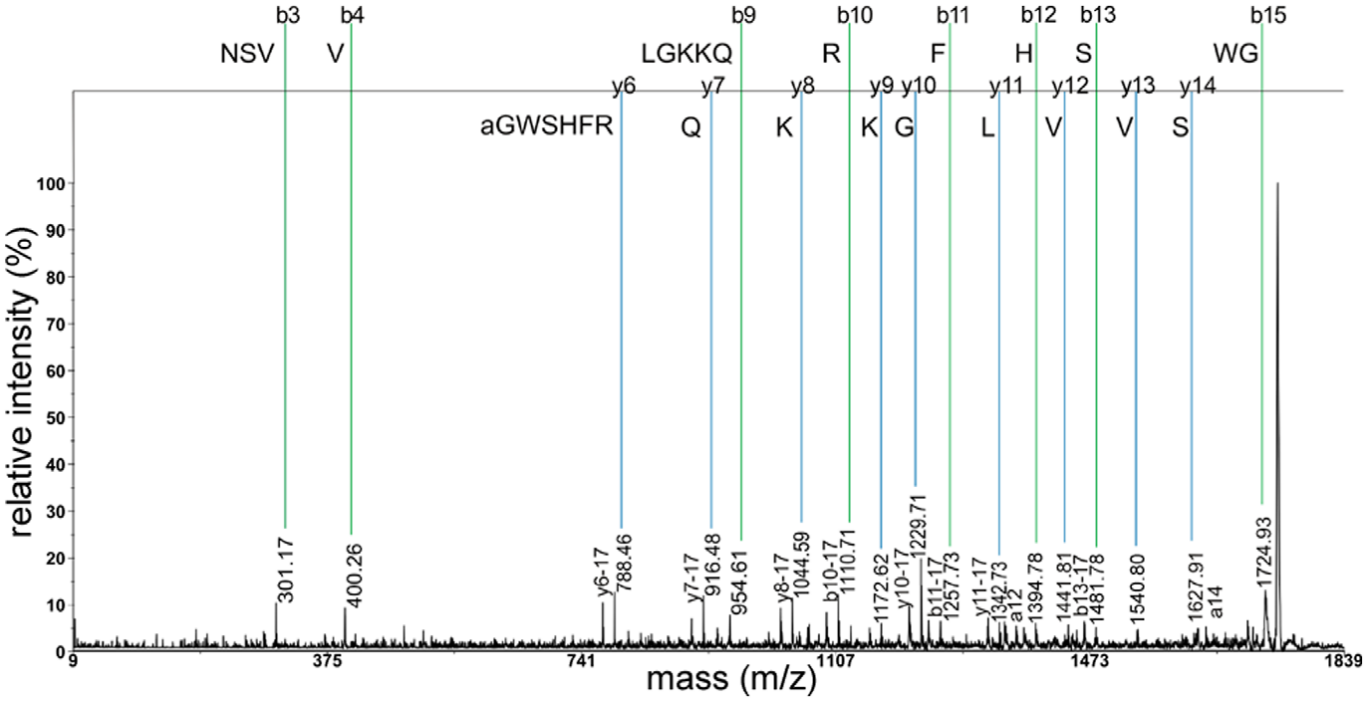
MS/MS PSD spectrum of kinin, obtained by direct profiling of a piece of the ventral ganglion.

For identification and sequence analysis of peptides from the larval CNS, we performed LC-MS/MS of a SPITC-labelled and an unlabeled extract of 40 CNS with attached ring glands. Peptides were then identified by aligning the measured fragmentation patterns with known peptides from *Drosophila* [25], [28] and adult *D. radicum* [9] as well as through manual *de novo* fragment annotation. The data revealed the presence of three A-type allatostatins, one C-type allatostatin with and without N-terminal pyroGlu, 10 FMRFa-like peptides, Yamide, CAPA-PK, CAPA-PK^2–15^, HUG-PK, CAPA-PVK-1 and -2, sNPF-1 and sNPF-1^4–11^, myosuppressin, myosuppressin^2–10^, SIFamide, sulfakinin, sulfakinin^6–14^, corazonin and corazonin^3–11^, two tachykinin-related peptides and a peptide very similar to *Drosophila* APK [28]. The sequences of these peptides are given in Table 1, the fragmentation spectra are shown in Fig. S2, S3, S4, S5, S6, S7, S8, S9, S10, S11, S12, S13, S14, S15, S16, and S17. Since sequences of *D. radicum* prepropeptide genes or ESTs are not available, it is difficult to rationally assign numbers for the different paracopies of the multicopy peptide families AST-A, tachykinin-related peptides and FMRFamides. As a neutral system, we therefore refer to the peptides with their mass as index (e.g. AST-A_909_ instead of AST-A-1). Instead of the typical C-terminal sequence PRVa, CAPA-PVK-2 from *D. radicum* ends on PRLamide, which has hitherto only been observed in the closely related flesh fly *Neobellieria bullata* as well as locusts (see [29]). Additionally, we also yielded the sequence of a kinin from direct peptide profiling and fragmentation of ventral ganglion fragments (Fig. 2). This kinin is sequence-identical to the kinin of *Drosophila* species [30].

### Peptides from the Midgut

Two LC-MS/MS runs of peptide extracts from 20 and 25 *D. radicum* larval midguts respectively led to the identification of four A-type allatostatins, one C-type allatostatin occurring with and without N-terminal pyroGlu, two tachykinin-related peptides and CCHamide1 (Table 1). AST-A_909_ and AST-A_953_, the AST-C and the two tachykinins had also been detected in the CNS by LC-MS/MS. AST-A_921_ has been found in the CNS by Audsley and colleagues [9]. All but one midgut peptide can thus be classified as brain-gut peptides. CCHamide1 was exclusively detectable in the midgut, but represents a brain-gut peptide in *Drosophila melanogaster* [31] and may have escaped detection in the *D. radicum* CNS.

### Direct Peptide Profiling and Fragmentation of Peptide Hormones from Neurohemal Organs

**Figure 3:**
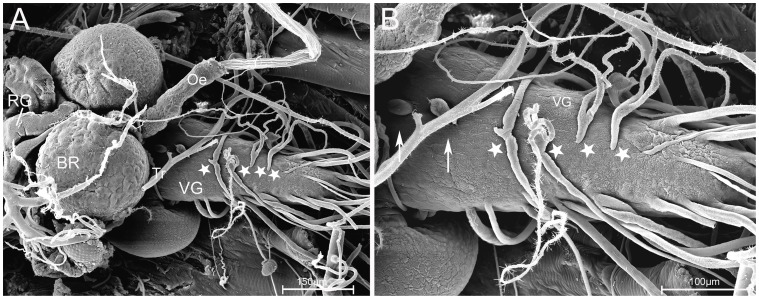
Morphology of the neurohemal organs of a *D. radicum* larva, scanning electron microscopy. A) Dorsal view of the larval nervous system consisting of the two brain hemispheres (BR) and the ventral ganglion (VG, to the right) comprising the suboesophageal, thoracic and abdominal neuromeres. The ring gland (RG) is visible to the left, attached to the brain. The stars mark the abdominal PSOs. B) Enlarged picture of the ventral ganglion. Four abdominal PSOs (stars) are visible as thickenings of the median/transverse nerve. Also two blindly-ending thoracic PSOs (arrows) are visible. Oe  =  oesophagus, Tr  =  trachea.

To identify potential neuropeptide hormones among the characterised peptides, we performed direct peptide profiling of isolated neurohemal tissues from individual larvae. The neurohemal organs associated with the CNS are the major source of neuropeptide hormones in insects. They consist of the corpora cardiaca (CC, containing terminals of secretory neurons with somata in the pars lateralis and pars intercerebralis), and the thoracic and abdominal perisympathetic organs (PSOs, containing terminals of secretory neurons with somata in the thoracic and abdominal neuromeres respectively). The CC also comprise an endocrine compartment containing the adipokinetic hormone (AKH)-producing cells. Scanning electron microscopy shows that the morphology of these organs in *D. radicum* larvae is typical for a cyclorrhaphan (Fig. 3): the CC are fused with the corpora allata and prothoracic gland and form a ring gland (Fig. 3A). Each thoracic neuromere shows a blind-ending thoracic PSO at its dorsal surface as also shown for *Drosophila* and *Calliphora* [32], [33]. Unlike *Drosophila*, however, *D. radicum* appears to have four instead of three abdominal PSOs, visible as swellings of the median/transverse nerves (Fig. 3B).

**Figure 4:**
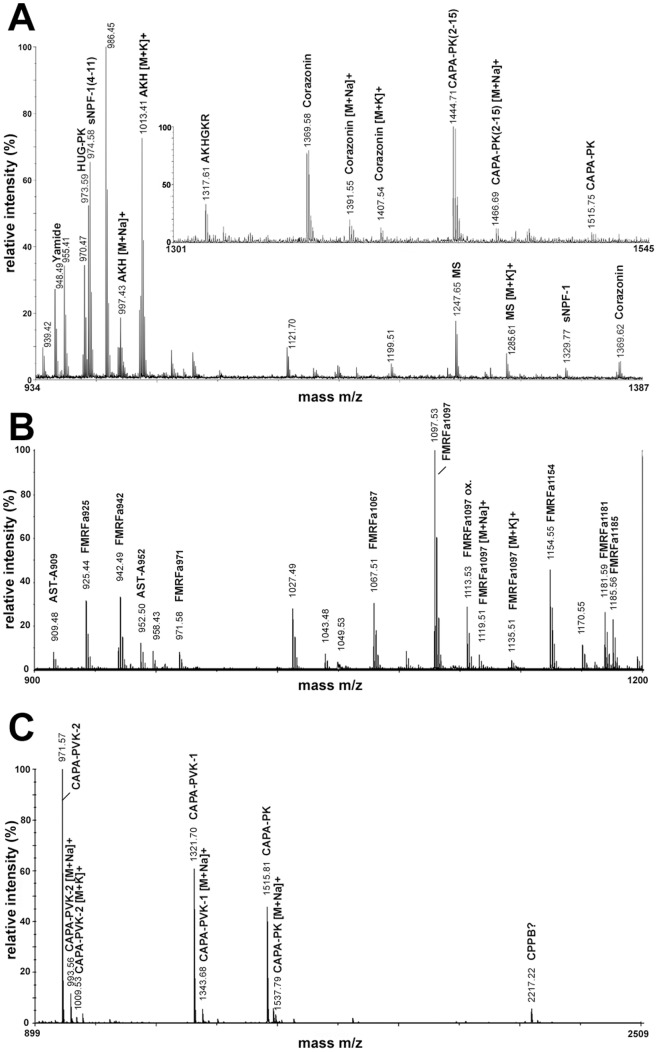
Typical spectra from direct peptide profiling. (A) Profile of the larval ring gland, (B) the anterior (thoracic), and (C) posterior (abdominal) portion of the adult dorsal sheath of the TAG. For some peptides, sodium and potassium adducts are visible besides the typical [M+H]^+^ adducts.

Earlier studies in *Drosophila* and other flies showed that direct mass spectrometric profiling of neurohemal organs leads to specific extraction and detection of peptides, while non-peptidergic signals are largely absent (e.g. [24], [25], [30], [34], [35]). A typical direct profile of a larval ring gland is shown in Figure 4A. The masses of 948.5 Da, 974.5 Da, 997.4/1013.4 Da, 1247.6 Da, 1329.8 Da and 1369.6 Da correspond to Yamide, sNPF-1^4–11^, AKH (Na^+^ and K^+^ adduct), myosuppressin (MS), sNPF-1 and corazonin from the ring gland of various *Drosophila* species [25], [30]. *D. radicum* CAPA-PK^2–15^ (1444.7 Da) and, with much less intensity, CAPA-PK (1515.2 Da) were consistently abundant. Also the AKH processing intermediates AKHGK (1161.6 Da) and AKHGKR (1317.6 Da) could consistently be detected, as well as *D. radicum* HUG-PK (973.6 Da) previously identified by Audsley and colleagues [9] in the adult CC. Subsequent direct PSD/CID fragmentation confirmed the identity of these peptides and the sequence data obtained by LC-MS/MS of CNS extracts (see Table 1). Further consistently detected masses were 939.4 Da, 955.4 Da, 1121.6 Da, 1125.5 Da, 1141.5 Da, 1143.6 Da and 1259.6 Da. None of these masses could be sequenced by direct fragmentation. The monoisotopic peak distribution however suggests that these masses represent peptides which thus remain to be characterised.

The PSOs are very small structures that are very difficult to separate from the larval CNS. Their homolog in adult cyclorrhaphan flies is the dorsal sheath of the adult thoracico-abdominal ganglion (TAG) [32], [33], [36], [37], [38], [39] which is much easier to dissect. A typical profile of this adult dorsal sheath is shown in Figure 4B (anterior “thoracic” region) and Figure 4C (posterior “abdominal” region).

The profiles of the thoracic region showed many different mass peaks, most of which corresponded to FMRFa-like peptides, while the posterior region is enriched in masses corresponding to CAPA peptides. With the exception of FMRFa_899_, all FMRFamide-like peptides identified by LC-MS/MS in whole CNS extracts could also be detected in the thoracic dorsal sheath preparation. This may suggest that FMRFa_899_ represents a degradation or processing intermediate of FMRFa_1185_. A consistent mass peak of 971.5 Da indicates the presence of FMRFa_971_ (EQDFMRFa) reported from adult *D. radicum* [9]. This peptide had not been found by LC-MS and could not be fragmented. Also APSQDFMRFa with an oxidised mass of 1113.5 Da characterised by Audsley *et al.* [9] from adult cabbage root flies was not found by LC-MS/MS of CNS extracts. However, a matching mass peak consistently occurred in direct profiles of the thoracic preparation but could not be fragmented - it may thus equally well represent the oxidised form of the mass-identical TPGQDFMRFa ( = FMRFa_1097_). The peaks corresponding to FMRFa_1097_ and FMRFa_1154_ gave higher signal intensities than other FMRFa-like peptides, suggesting that the peptides are encoded in three and two copies in the *fmrf* prepropeptide gene respectively (e.g. [24], [25]). Alternatively, if APSQDFMRFa also occurs in the larva albeit undetected, the peak at 1097.6 Da represents the integrated intensity of both APSQDFMRFa and TPGQDFMRFa.

The profiles of the abdominal dorsal sheath preparation only showed four peaks, corresponding to CAPA-PVK-1 and -2, CAPA-PK and a mass of 2217.2 Da. The same preparation in other fly species show also three CAPA peptides [24], [34] plus -at least in *Drosophila* species- a non-amidated cleavage product in the 2200 Da range (CAPA precursor protein B (CPPB)) [25], [30]. While a direct fragmentation could not be achieved, it is therefore likely that the peak at 2217.2 Da represents the CPPB of *D. radicum*.

### Distribution Pattern of Peptidergic Cells

To compare the general cellular architecture of peptidergic systems in *D. radicum* larvae with that of other flies, we performed immunofluorescent stainings with a host of peptide antisera.

#### Peptidergic neurons in the CNS and ring gland

**Figure 5:**
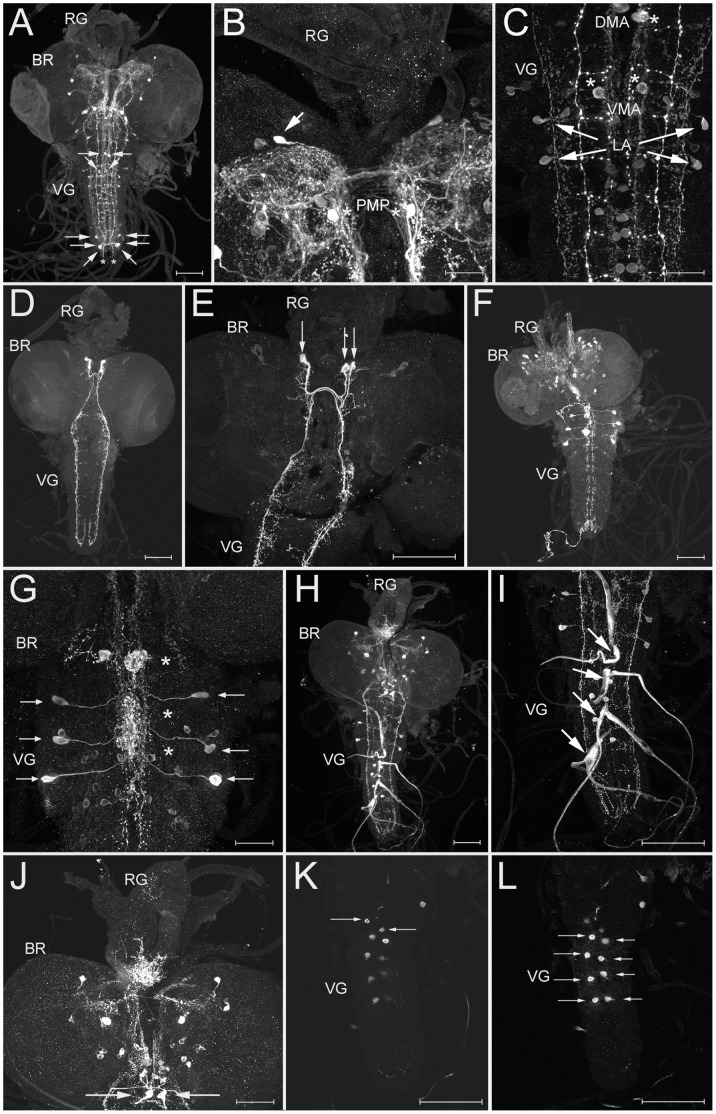
Wholemount immunostainings of the larval CNS. A-C) AST-A immunoreactivity. A) Dorsal overview (maximum projection). Arrows point to prominently stained somata in the ventral ganglion. Exiting neurites innervating the hindgut are marked by an asterisk. B) Detail of the dorsal protocerebrum showing a pair of strongly labelled PMP-like neurons (asterisks) and a strongly labelled neuron not described in *Drosophila* (arrow). C) Detail of the metathoracic and first abdominal neuromeres, showing the strongly labelled MA (asterisks) and LA (arrows) neurons, and further very weakly stained cells at the midline. D-E) SIFa immunoreactivity. D) Dorsal overview (maximum projection) of the two pairs of SIFa neurons in the protocerebrum, that send contralaterally descending fibres through the ventral ganglion. E) Close-up showing the arborisation pattern of the descending fibres. Somata are marked by arrows. F-G) FMRFa-like IR. F) Dorsal overview (maximum projection) of the whole CNS. Immunoreactive somata are most prominent in the dorsal protocerebrum and the thoracic neuromeres. G) Detail of the thoracic neuromeres. The thoracic PSOs are strongly stained in a varicose fashion likely due to stored peptide vesicles (asterisks). A pair of strongly stained Tv neurons is visible in each neuromere (arrows). H-L) PRXa-like IR. H) Dorsal overview (maximum projection) of the whole CNS. Immunoreactive somata are most prominent in the dorsal protocerebrum, suboesophageal ganglion and the anterior abdominal neuromeres. I) Detail showing the ventral ganglion with four pairs of strongly stained median/transverse nerves in the anterior abdominal neuromeres. The swellings along the median and transverse nerves represent the abdominal PSOs (arrows). J) Detail showing the brain, ring gland and suboesophageal ganglion. Somata in the protocerebrum are visible. The strong immunoreactivity in the ring gland is due to innervation by the CC-MS neurons (arrows) in the suboesophageal ganglion. K) Detail showing the ventral ganglion with a pair of strongly stained neurons (arrows) anterior to the Va neurons. This pair seems to be a different cell type than the Va neurons due to differences in shape and the lack of neurohemal projections. L) Detail showing the ventral ganglion with four pairs of strongly stained Va neurons (arrows) in the anterior abdominal neuromeres. BR  =  brain, RG  =  ring gland, VG  =  ventral ganglion. Scale bars  = 150 µm, in B, C, G and J  = 50 µm.

*AST-A IR:* Clusters of AST-A IR cell bodies and descending neurites are prominent in the brain and ventral ganglion (Fig. 5A-C), and are highly similar in number and morphology to the bilateral pairs of AST-A PMP, LP and LT neurons in the brain, and the DMA, VMA, LA and LAa neurons in the ventral ganglion of larval *Drosophila* [40], [41]. *D. radicum* has, however, further pairs of AST-A IR brain neurons, e.g. in the posterior protocerebrum (Fig. 5B). Like in *Drosophila*, the LAa neurons send neurites to the hindgut through segmental nerve 8/9 and the ring gland is devoid of AST-A IR; neurites projecting towards the ring gland could not be detected (Fig. 5A). Also in adult *Calliphora*, posterior AST-A IR LAa-like neurons innervate the hindgut, and the CC are devoid of AST-A IR [42].

*SIFamide IR:* Two pairs of strongly SIFamide-immunoreactive somata are located in the pars intercerebralis (Fig. 5D-E). Their axons project to contralateral parts of the protocerebrum and descend through the entire ventral ganglion. Thus, the pattern is identical to that of the larval SIFamide neurons in *Drosophila melanogaster* and adult *Neobellieria (Sarcophaga) bullata* [17], [41] and other insects [17].

*FMRFamide IR:* The used antiserum recognises not only FMRFamide-like peptides, but (less strongly) also other peptides with a C-terminal RFamide (sNPF, sulfakinin, myosuppressin, neuropeptide F). Relatively weak FMRFamide IR is visible in the ring gland and in bilaterally symmetric somata in the pars intercerebralis (Fig. 5F-G). Since sNPF-1 and myosuppressin are the only RFamides found via mass spectrometry in the ring gland, at least part of this IR is likely to be attributable to sNPF-containing secretory or myosuppressin neurons. A pair of strongly stained secretory neurons is visible in each of the three thoracic neuromeres (Fig. 5G). These neurons innervate the thoracic PSOs and appear to be homolog to the FMRFamide-like peptide expressing Tv neurons of *Drosophila melanogaster* [41], [43]. Similar neurons have also been described in larvae of *Lucilia cuprina* [35], *Sarcophaga bullata* [39] and *Calliphora erythrocephala* [33].

*PRXamide IR:* PRXamide IR labels pyrokinins and periviscerokinins ending on either PRLamide or PRVamide [21]. Prominent PRXamide IR is visible in the CC part of the ring gland, and the abdominal PSOs/transverse nerves 1–4 (Fig. 5H-L). The MS data and the situation in *Drosophila melanogaster* [25] indicates that the ring gland staining represents HUG-PK and CAPA-PK^2–15^, while the aPSO staining represents both CAPA-PK and CAPA-PVKs. Large neurosecretory cells (Fig. 5J) in the suboesophageal neuromeres -likely homologs of the hugin-expressing CC-MS-1 and capa-expressing CC-MS-2 cells of *Drosophila* [44], [45], [46]- provide the immunoreactivity of the ring gland. Each of the four abdominal PSOs (see Fig. 3) is innervated by a pair of neurons homolog to the Va neurons of *Drosophila* (Fig. 5K-L, [47]). The number of Va neuron pairs thus matches that of abdominal PSOs like in *Drosophila melanogaster* larvae which, however, only have three PSOs and Va neuron pairs respectively [32].

**Figure 6:**
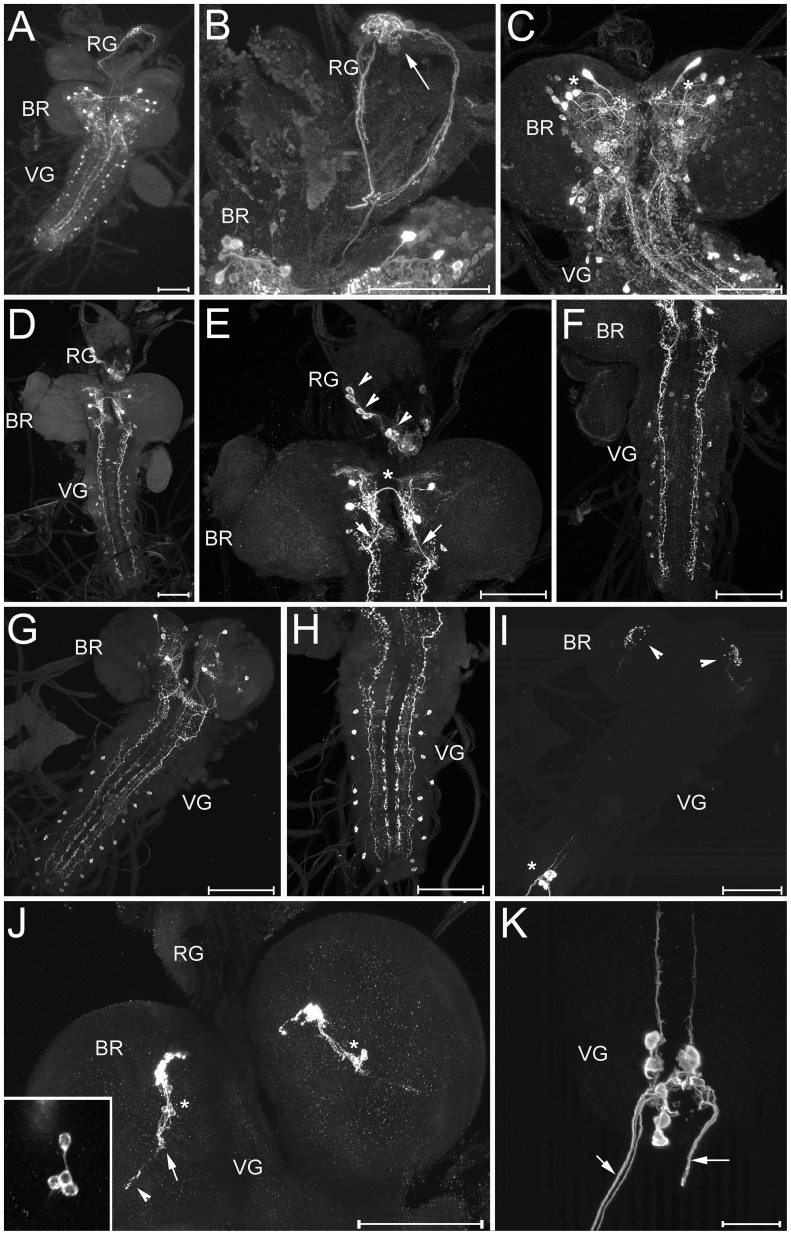
Wholemount immunostainings of the larval CNS. A-C) DH31 immunoreactivity. A) Dorsal overview (maximum projection). Large strongly stained somata in the protocerebrum and further smaller cells are visible, as well as paired lateral neurons in the ventral ganglion. B) Detail of the protocerebrum and ring gland. Several DH31-immunoreactive fibres project over the whole ring gland and branch intensely around the corpora allata (arrow). C) Close-up of the brain and suboesophageal neuromeres shows that many neurons are DH31-immunoreactive. The most strongly stained neurons in the dorsal protocerebrum (asterisks) may represent the neurosecretory neurons innervating the ring gland, but their exact projection pattern could not be singled out from the dense arborisations. D-F) MIP immunoreactivity. D) Dorsal overview (maximum projection). E) Close-up of the brain and ring gland. Two pairs of large and strongly stained neurons in the brain are visible. While the more dorsal pair appears to send a neurite to the contralateral brain hemisphere (asterisk), the more ventral pair seems to give rise to the descending bilateral fibre (arrows) that projects through the whole ventral nerve cord. Even more ventral in the brain, a pair of smaller neurites is stained. Their processes could not be followed. In the ring gland, intrinsic cells in the corpora cardiaca are labelled (arrow heads). These cells most likely do not produce MIP but are cross-reacting AKH endocrines. F) Close-up of the ventral ganglion. The descending fibres with small branchings and varicosities along their track are visible. One pair of lateral cells in the thoracic and abdominal neuromeres are also stained. G-H) Tachykinin-like peptide immunoreactivity. G) Dorsal overview (maximum projection) and H) magnification of the ventral ganglion. Note the absence of stained somata in the suboesophageal ganglion. I-K) PDF-like immunoreactivity. I) Dorsal overview (maximum projection). The lateral neurons (LNs) in each brain hemisphere with strongly stained arborisations in the dorsal protocerebrum (arrowheads), and the abdominal neurons (asterisk) with neurites projecting to the hindgut are visible. J) Close-up of the two brain hemispheres, each with a group of LNs (asterisks) sending neurites to the dorsal protocerebrum and to the putative larval optic neuropile (arrow). Interestingly, some neurites appear to project beyond the optic neuropile (arrowhead), possibly along the entering optic (“Bolwig”) nerve. The inset shows a magnification of the four LNs. K) Close-up of the PDF-neurons in the last abdominal neuromeres which send neurites through the last segmental nerves towards the hindgut (arrows) BR  =  brain, RG  =  ring gland, VG  =  ventral ganglion. Scale bars  = 150 µm, in K  = 50 µm.

*Diuretic hormone-31 (DH31) IR:* The antiserum against DH31 stained a complex pattern of somata with broad arborisations in both the brain and ventral ganglion (Fig. 6A-C). Again the overall pattern was very similar to that described in the *Drosophila melanogaster* maggot [48]. The ring gland is innervated by DH31-positive neurites that most likely originate from somata in the pars intercerebralis. Unlike most peptidergic terminals that end in the CC part, these DH31-immunoreactive neurites end in a neurite meshwork in the region of the corpora allata. This opens the possibility that DH31 may play a role in the regulation of juvenile hormone synthesis or release.

*Myoinhibiting peptide (MIP) IR:* The pattern of MIP immunoreactivity in the brain and ventral ganglion (Fig. 6D-F) was again strongly reminiscent to the situation in *Drosophila melanogaster* [49]. Strongly stained neurons and descending neurites are prominent in the brain (Fig. 6D). In the protocerebrum, one pair of neurites projects contralaterally dorsal to the foramen (Fig. 6E). One pair of median cells in the suboesophageal neuromeres, and a pair of lateral cells in the thoracic and all but the last abdominal neuromeres are stained (Fig. 6F). In larval *Drosophila melanogaster,* similar cells express also CCAP [50]. MIP immunoreactivity also occurred in intrinsic endocrine cells of the glandular CC part of the ring gland (Fig. 6E). Since MIPs could not be detected in the ring gland by mass spectrometry in *D. radicum* and other fly species [9], [25], [30], [51], and since in all insects the intrinsic endocrine cells produce AKH, it seems unlikely that the MIP IR in the ring gland represents the occurrence of MIPs. The antiserum recognises the C-terminus of Pea-MIP (GGWamide), and we thus rather assume a cross-reaction with AKH (ending Wamide) in the ring gland, while the staining in the CNS is more likely to be MIP-specific.

*Tachykinin-related peptide (TK) IR:* Bilaterally symmetric TK-immunoreactive cells are situated in both brain and ventral ganglion (Fig. 6G-H). No TK-IR was observed in neurohemal organs. Again, the number, pattern of somata and projections is very similar to the situation in *Drosophila* [19], [52] and also the blowfly *Calliphora vomitoria* [53], with prominent descending neurites originating in the brain and running along a lateral tract throughout the ventral ganglion. Unlike in *Drosophila* and *Calliphora*, however, TK-immunoreactive somata were not discernible in the suboesophageal ganglion.

**Figure 7:**
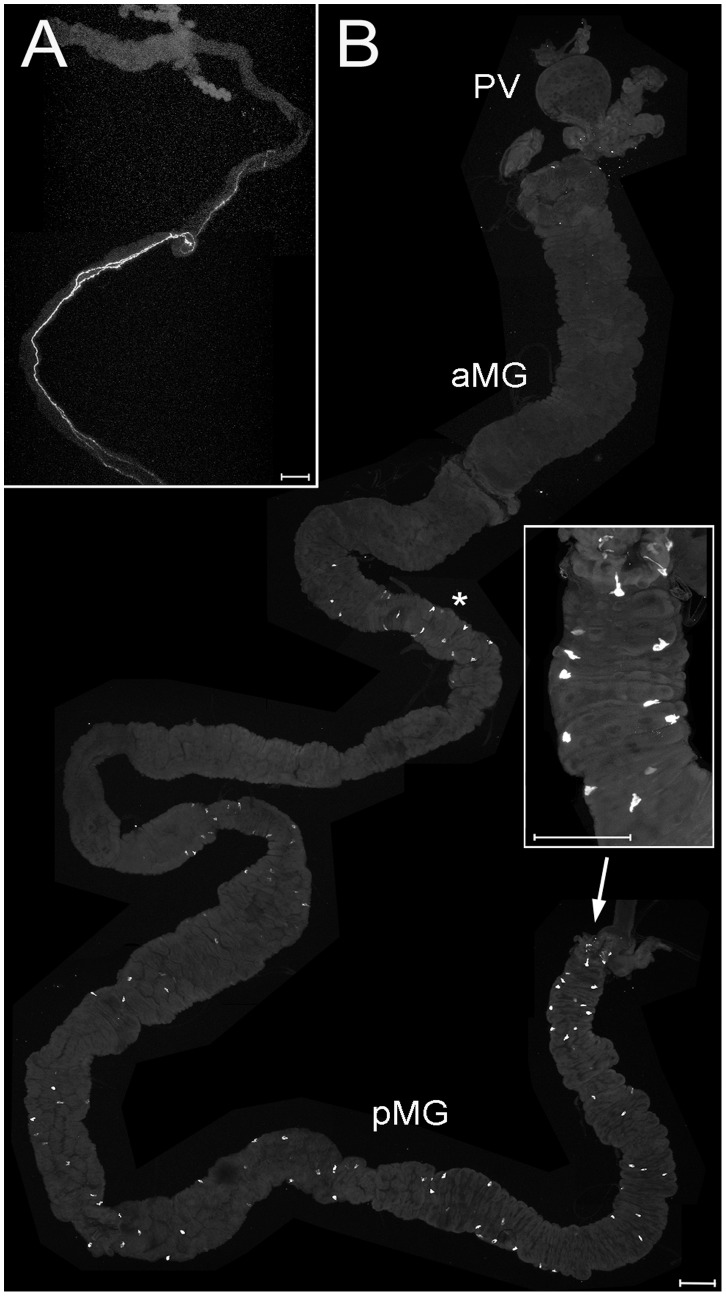
PDF and tachykinin-like immunoreactivity in the larval gut. A) PDF-immunoreactive neurites innervate the hindgut, but do not reach the midgut or Malpighian tubules. B) Tachykinin-like-immunoreactive EECs are visible in a region possibly representing the anterior-middle midgut junction (asterisk). Further immunoreactive cells are scattered throughout the posterior midgut. The most posterior midgut portion is shown enlarged in the inset. PV  =  proventriculus, aMG  =  anterior midgut, pMG  =  posterior midgut. Scale bars  = 150 µm.

*Pigment dispersing factor (PDF) IR:* Two different clusters of PDF immunoreactive neurons could be observed (Fig. 6I-K). One cluster of four cells is located in each half of the brain, sending projections to the dorsal protocerebrum and to the putative larval optic neuropile (Fig. 6J). The number, morphology and PDF IR identify these cells as homologs of the lateral neurons (LNs) of larval *Drosophila melanogaster* [54]. The second group of PDF-immunoreactive cells is located in abdominal neuromeres 8 and 9, again identical to the situation in *Drosophila melanogaster* [41], [54]. Like in the fruit fly [54], their axons exit the ventral ganglion through segmental nerve a8 and innervate the hindgut (Fig. 6K, Fig. 7A). Both LNs and the abdominal neurons also occur in the housefly, though additional PDF-immunoreactive neurons neither found in *Drosophila melanogaster* nor *D. radicum* have been described for larval *Musca domestica* [55].

#### Peptidergic enteroendocrine cells

Tachykinin-like-immunoreactive enteroendocrine cells (EECs) occurred in a region possibly presenting the anterior-middle midgut junction, and scattered throughout the posterior midgut (Fig. 7B). In larval *Drosophila melanogaster*, tachykinin-like immunoreactive EECs have only been found in the posterior midgut; more anterior parts seem to be devoid of tachykinin-like IR [52], [56]. Both in *Drosophila* and *D. radicum* larvae, the highest density of Tk-IR cells in the posterior midgut is seen in the short portion closest to the hindgut.

**Figure 8:**
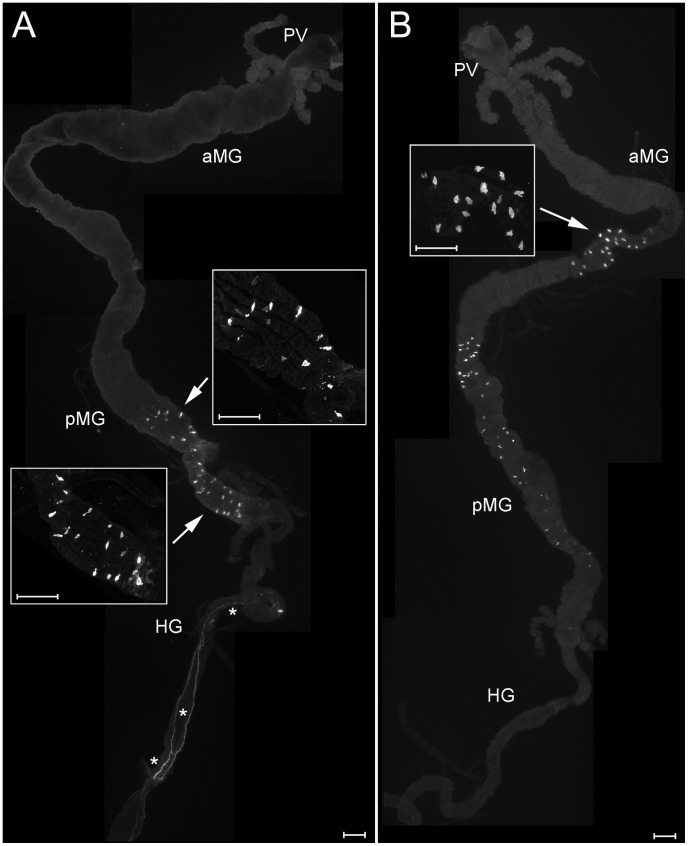
AST-A and MIP (AST-B) immunoreactivity in the larval gut. A) AST-A immunoreactive EECs are located in the posterior midgut, their typical tear-drop like shape is visible in the insets. On the hindgut, AST-A immunoreactive neurites are labelled (asterisks). B) MIP-immunoreactive EECs are densely located in the anterior middle midgut (inset), smaller cells are visible throughout the posterior middle and posterior midgut. PV  =  proventriculus, aMG  =  anterior midgut, pMG  =  posterior midgut, HG  =  hindgut. Scale bars  = 150 µm.

Like in *Drosophila melanogaster* [40], AST-A-immunoreactive EECs are located in the posterior midgut, and AST-A-IR neurites from the CNS innervate the hindgut (Fig. 8A). The AST-A-IR EECs are apically elongated and teardrop-shaped, thus reminiscent of the typical structure of open type EECs (Fig. 8A).

Strongly stained myoinhibitory peptide (MIP)-IR cells are densely located in a relatively short midgut portion possibly representing the anterior-middle midgut junction (Fig. 8B). Thus, these cells are another common attribute of *D. radicum* and *Drosophila* larvae. Smaller MIP-IR EECs occur in the middle and posterior midgut, whereas in *Drosophila* these portions of the gut show only weak MIP immunoreactivity.

**Figure 9:**
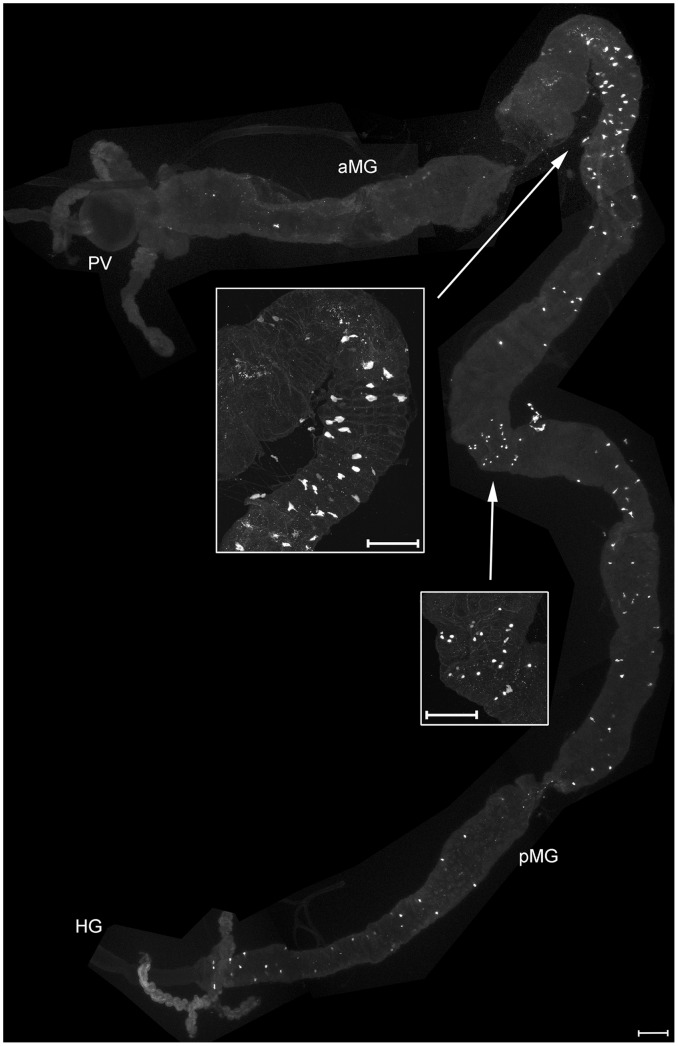
DH31 immunoreactivity in the larval gut. Parts of the midgut containing stained EECs are enlarged in the insets. DH31-immunoreactive EECs are located throughout the middle and posterior midgut, and are also densely distributed around the presumptive anterior-middle midgut junction. PV  =  proventriculus, aMG  =  anterior midgut, pMG  =  posterior midgut, HG  =  hindgut. Scale bars  = 150 µm.

Numerous diuretic hormone 31 (DH31)-IR EECs are located in the posterior portion of the anterior midgut, where they are largest and show open EEC type-like cytoplasmic extensions (Fig. 9). Smaller and more roundish DH31-IR cells are also found in the middle midgut and posterior midgut (Fig. 9).

In general, the pattern of immunoreactivity in the larval midgut is very similar to that described in *Drosophila melanogaster* for AST-A [40], [56], while smaller differences occur for TK-, MIP-and DH31-immunoreactive EECs. Like in the fruit fly larva [56], also the larval *D. radicum* hindgut is innervated by PDF-immunoreactive neurites which do not reach the midgut (Fig. 7A).

### Yamide is also Present in Drosophila Species and Represents an Eclosion Hormone-related Peptide

**Figure 10:**
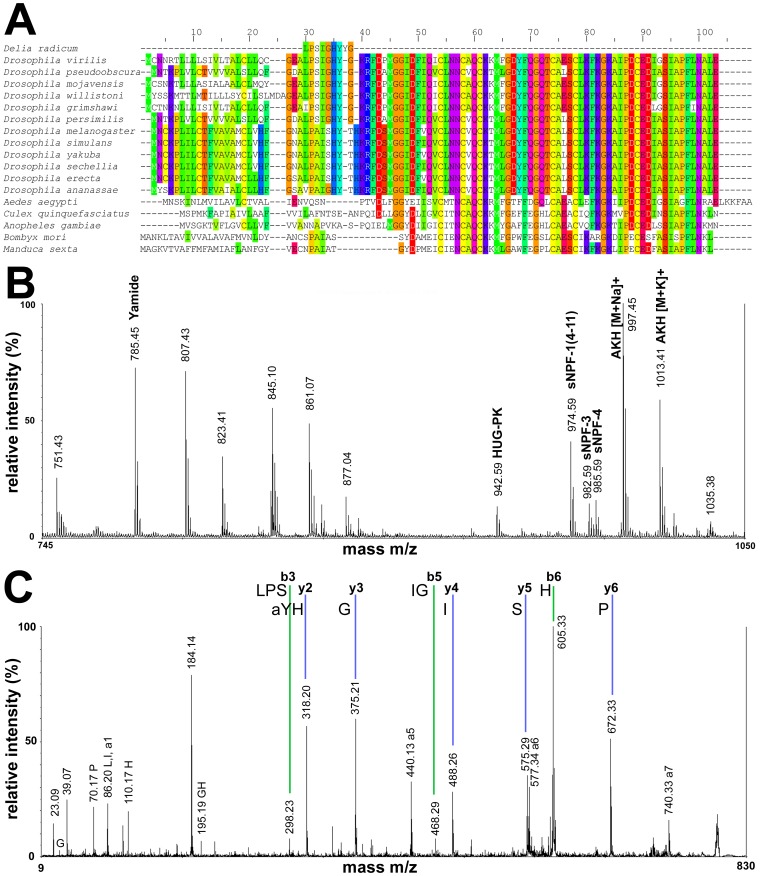
Yamide in Dipterans and moths. A) Alignment of the eclosion hormone prepropeptides of different fly, mosquito and moth species with *D. radicum* Yamide (aligned at position 30), generated with Jalview 2 [77]. The predicted signal peptide cleavage site locates at aligned position 29. In all *Drosophila* species, the Yamide-aligning sequences are followed by a dibasic cleavage site (KR, at aligned position 40), which is absent in mosquitoes and moths. An amidation signal (G) precedes this cleavage sites in fruitflies outside the *melanogaster* group, while the flies of the *melanogaster* group possess the sequence TH instead. B) Direct mass spectrometric profile of the ring gland of a wandering third instar larva of *Drosophila virilis*. Several mass peaks are visible, which above 900 Da represent known neuropeptides. The mass peak at 785.45 corresponding to *Drosophila virilis* Yamide typically showed a high relative intensity comparable with that of the abundant sNPF-1^4–11^ and AKH peptide hormones. C) Combined post-source and collision-induced decay spectrum of the mass peak at 785.45 Da reveals the identity of *Drosophila virilis* Yamide.

C-terminal amidation is a unique modification of regulatory peptides, and is generated from a C-terminal glycine residue by a specific set of enzymes occurring in peptidergic cells [27]. Peptides originating from the break-down of proteins therefore do not carry an amidation signal. The presence of a Yamide signal in direct profilings of the neurohemal ring gland suggests that this peptide is released as a neurohormone, while its amidation may suggest bioactivity. Since peptides are evolutionarily strongly conserved, it would be surprising if Yamide, a peptide without published homologs in other insect species, only occurred in *D. radicum*. An unrestricted blast search based on the sequence LPSIGHYYG identified a highly similar sequence only for *Drosophila* species outside the *melanogaster* group (Fig. 10A). The identified sequence in the non-*melanogaster* fruitflies represents a short peptide stretch of the respective eclosion hormone precursor. This stretch is N-terminally joined to the signal peptide, and C-terminally extended by KR, the processing signal for prohormone convertases. It is also present in the EH prepropeptide of *melanogaster* fruitflies and the relatively closely related mosquitoes and moths, yet with a differing sequence and without an amidation signal (Fig.10A). This predicts that for the *Drosophila* species outside the *melanogaster* group, a Yamide is produced during the normal processing of eclosion hormone. In *Drosophila*, eclosion hormone is stored and released from the ring gland, which predicts that also Yamide should be stored in this neurohemal organ. To test this, we directly profiled the ring gland of wandering L3 larvae of *Drosophila virilis* by MALDI-TOF MS. In all preparations (n = 16), a prominent peak of the predicted mass 785.43 Da was detectable (Fig. 10B). Tandem MS of this peak yielded a complete fragmentation spectrum indicating the sequence LPSIGHYa and thus confirming the presence of Yamide in *Drosophila virilis* (Fig. 10C).

## Discussion

We have chemically characterised 38 peptides (including variants of different size and N-terminal pyroglutamination) from the nervous system, neurohemal organs and midgut of larval *D. radicum*. Of these peptides, sNPF [57], HUG-pyrokinin [58], kinins [59], [60], [61], [62], CAPA-PVKs [44], [63], AST-A [64] and AKH [65], [66], [67] have important effects on feeding and diuresis in *Drosophila* and other Dipterans. Since it is likely that the function of these peptides is conserved within the Diptera, their signalling pathways are potential targets for a chemical control of *D. radicum* larvae. The available peptide sequence data for *D. radicum* maggots and adults now allow physiological and pharmacological studies with native peptides in this species, and may possibly provide a platform for the future development of peptide-based protectants against cabbage maggot infestation.

### The SPITC Labelling Approach Strongly Improved MALDI-TOF/TOF De Novo Sequencing

Peptide fragmentation by mass spectrometry has largely substituted traditional peptide sequencing methods since it in principle allows *de novo* sequencing of peptides from very little material. A caveat for MALDI-based mass spectrometric peptide fragmentation is that the obtained fragmentation patterns can be very complex due to the many different types of fragments that are generated. These include N- and C-terminal fragments, immonium ions and internal fragments, sometimes accompanied by satellite peaks caused by the loss of water or ammonia. In species with sequenced genome this is rarely a problem, since the whole fragment spectrum can be predicted from the respective candidate gene. Not surprisingly, most insect species with characterised peptidomes belong to those (still rather rare) species with a sequenced genome. Considerable peptidomic data for species without sequenced genome or EST data banks exist only for large insects such as cockroaches, locusts and blowflies for which enough peptide could be extracted for traditional Edman sequencing (see [68]) or *de novo* mass spectrometric sequencing [69], [70], [71]. Since SPITC labelling directs fragmentation towards y-fragments [12], [22], it strongly decreased the complexity of the PSD fragmentation pattern in this study. This allowed us to characterise a substantial (so clearly not the full) complement of peptides present in the comparatively small cabbage root fly larvae for which no genomic or suitable EST sequences are available. Our results confirm and extend previous results from adult cabbage root flies [9], suggesting that the neuropeptide complement does not change qualitatively between the maggot and adult fly. Also in *Drosophila*, the peptide complement does not change qualitatively during postembryonic development [24], [25], [28]. Nevertheless, our sequence data differ from those of Audsley and colleagues for FMRFa_1154_ (SAPGQDFMRFa vs. SPKQDFMRFa) and FMRFa_1181_ (LPEQDFMRFa vs. KPNQDFMRFa), contradictions which will be solved once the *D. radicum fmrf* gene sequence is available. The FMRFamide-like peptides represent the most variable group of insect neuropeptides, and the available fly genes suggest a very high degree of internal variation [30], [72]. Moreover, strain-specific FMRFamide-like peptides have been reported for *Lucilia cuprina* [35]. Therefore, it is also possible that the different sequences obtained in this study (based on a laboratory strain originating from Germany) and the study of Audsley *et al.* [9] (based on a UK laboratory strain) are genuine and reflect genetic variation between separated populations.

### Immunostainings Confirm the Mass Spectrometric Data and Show that Peptide Families have been Missed by MS

The observed patterns of immunoreactivity for AST-A, SIFa, FMRFamide-like peptides, pyrokinins/periviscerokinins and tachykinin-like peptides confirm the peptide distribution found by direct peptide profiling of neurohemal organs and LC/MS of the CNS and gut. The immunostainings against PDF, MIP and DH31 show, however, that there are further *D. radicum* peptides which we were unable to characterise. Larger peptides such as insulins, eclosion hormone etc. are notoriously difficult for peptidomics and were also not found here. At least, however, PDF, DH31 and MIPs could be detected by LC/MS in *Drosophila melanogaster* [28], [31] and may be also detectable in *D. radicum* with improved chromatographic separation reducing ion suppression. Thus, while the available sequence for 38 peptides puts *D. radicum* on the list of the best characterised Dipterans in terms of peptides, there are certainly more peptides to be discovered in this species.

### D. radicum Shows Fly-typical Presence and Distribution of Peptide Hormones

In general, the peptide families identified in *D. radicum* are common in cyclorrhaphan fly species (e.g. [24], [25], [28], [30], [31], [35], [51], [71]), and the peptide hormone complement in neurohemal organs and enteroendocrine cells is typical for this fly group [24], [25], [30], [56], [31], [35], [34], [51]. Moreover, our anatomical results emphasise that the gross distribution and projection patterns of the immunostained peptidergic neurons and enteroendocrine cells in *D. radicum* is largely similar to that in *Drosophila melanogaster* and other less well investigated flies (see section 3.4), even though differences may occur in finer details (e.g. the dendritic projection patterns) which we have not studied here. This chemical and anatomical similarity of peptidergic systems between *D. radicum* and *Drosophila melanogaster* suggests that the fruit fly may well constitute a useful genetically amenable (neuro)endocrine model for cyclorrhaphan pest species besides its importance as a general developmental or neurobiological model organism. This is particularly evidenced by sequence-identical peptides in *Drosophila melanogaster, D. radicum* and other flies (e.g. sNPF-1, myosuppressin, SIFamide). Nevertheless, from a peptidomic perspective, *D. radicum* is clearly closer to the blowflies than to *Drosophila melanogaster*. For example, the sulfakinin and tachykinin sequences are much more similar to that of *Calliphora vomitoria* [73], [74] than *Drosophila melanogaster* [19], [28]. Thus, our sequence data is in support of the phylogenetically grouping of Anthomyiidae, Sarcophagidae and Calliphoridae within the Calyptratae, a sister group of the Ephydroidea (*Drosophila* and allies) [75].

In light of the above, the restricted occurrence of the Yamide in Anthomyiidae and *Drosophila*-species outside the *melanogaster* group is remarkable. Our mass spectrometric data show that Yamide is stored in higher concentrations in the ring gland, but this might simply be a consequence of its C-terminal glycine (absent in e.g. *Drosophila melanogaster*) which is amidated due to co-processing and co-packaging with eclosion hormone. We therefore assume that Yamide represents an evolutionary caprice without biological function, at least until a receptor for this peptide family has been identified.

## Supporting Information

Figure S1 **MS/MS spectrum of unlabeled AKH.**
(TIF)

Figure S2 **MS/MS spectrum of AST-A909, SPITC-labelled.**
(TIF)

Figure S3 **MS/MS spectrum of FMRFa885, SPITC-labelled.**
(TIF)

Figure S4 **MS/MS spectrum of FMRFa899, SPITC-labelled.**
(TIF)

Figure S5 **MS/MS spectrum of FMRFa996, unlabeled.**
(TIF)

Figure S6 **MS/MS spectrum of FMRFa1097 with an oxidised methionine (1113.5 Da), SPITC-labelled.**
(TIF)

Figure S7 **MS/MS spectrum of FMRFa1154, unlabeled.**
(TIF)

Figure S8 **MS/MS spectrum of FMRFa1181, SPITC-labelled.**
(TIF)

Figure S9 **MS/MS spectrum of FMRFa1185, SPITC-labelled.**
(TIF)

Figure S10 **MS/MS spectrum of CAPA-PK, SPITC-labelled.**
(TIF)

Figure S11 **MS/MS spectrum of CAPA-PVK-1, SPITC-labelled.**
(TIF)

Figure S12 **MS/MS spectrum of CAPA-PVK-2, SPITC-labelled.**
(TIF)

Figure S13 **MS/MS spectrum of SIFa, SPITC-labelled.**
(TIF)

Figure S14 **MS/MS spectrum of sulfakinin6-14, SPITC-labelled.**
(TIF)

Figure S15 **MS/MS spectrum of TK1116, SPITC-labelled.**
(TIF)

Figure S16 **MS/MS spectrum of TK1010, SPITC-labelled.**
(TIF)

Figure S17 **MS/MS spectrum of APK, SPITC-labelled.**
(TIF)
