# Supplementary figures and images for: Peptidomics of the Agriculturally Damaging Larval Stage of the Cabbage Root Fly Delia radicum (Diptera: Anthomyiidae)
Source: PLoS One. 2012 Jul 25;7(7):e41543. doi: 10.1371/journal.pone.0041543 (PMC3405134; doi:10.1371/journal.pone.0041543)

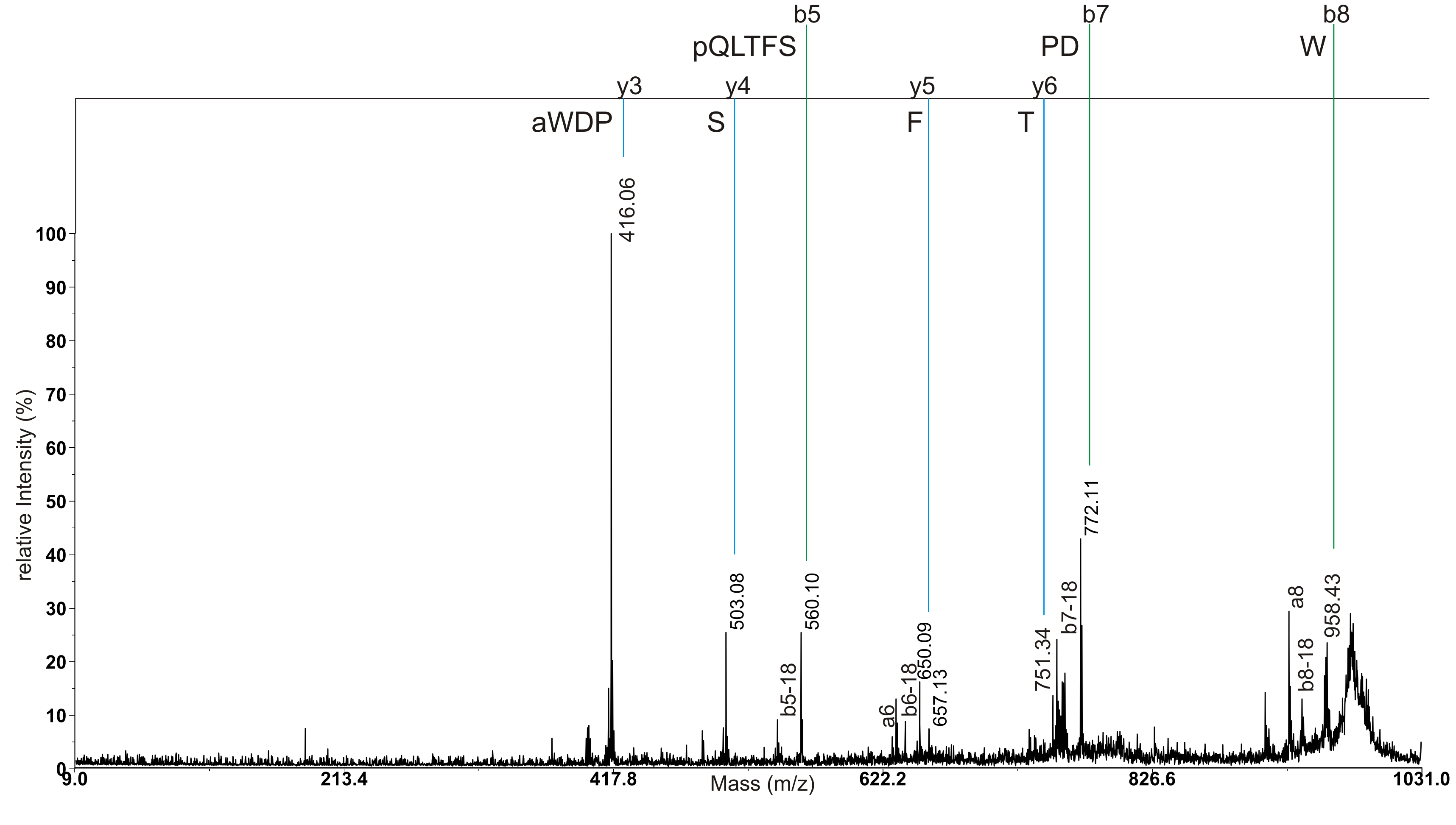

Supplement: Figure S1 — MS/MS spectrum of unlabeled AKH. (TIF) [file pone.0041543.s001.tif]

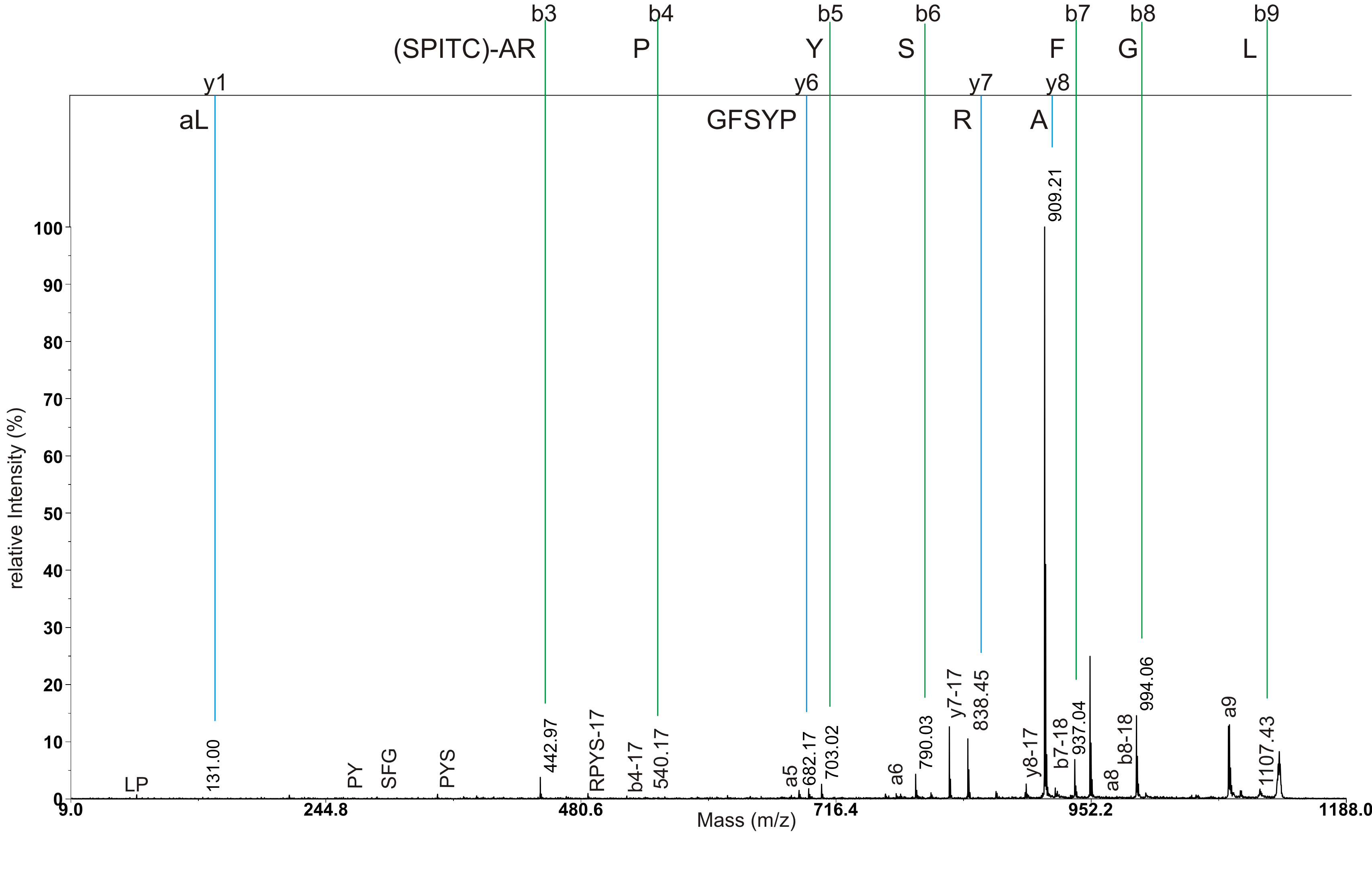

Supplement: Figure S2 — MS/MS spectrum of AST-A909, SPITC-labelled. (TIF) [file pone.0041543.s002.tif]

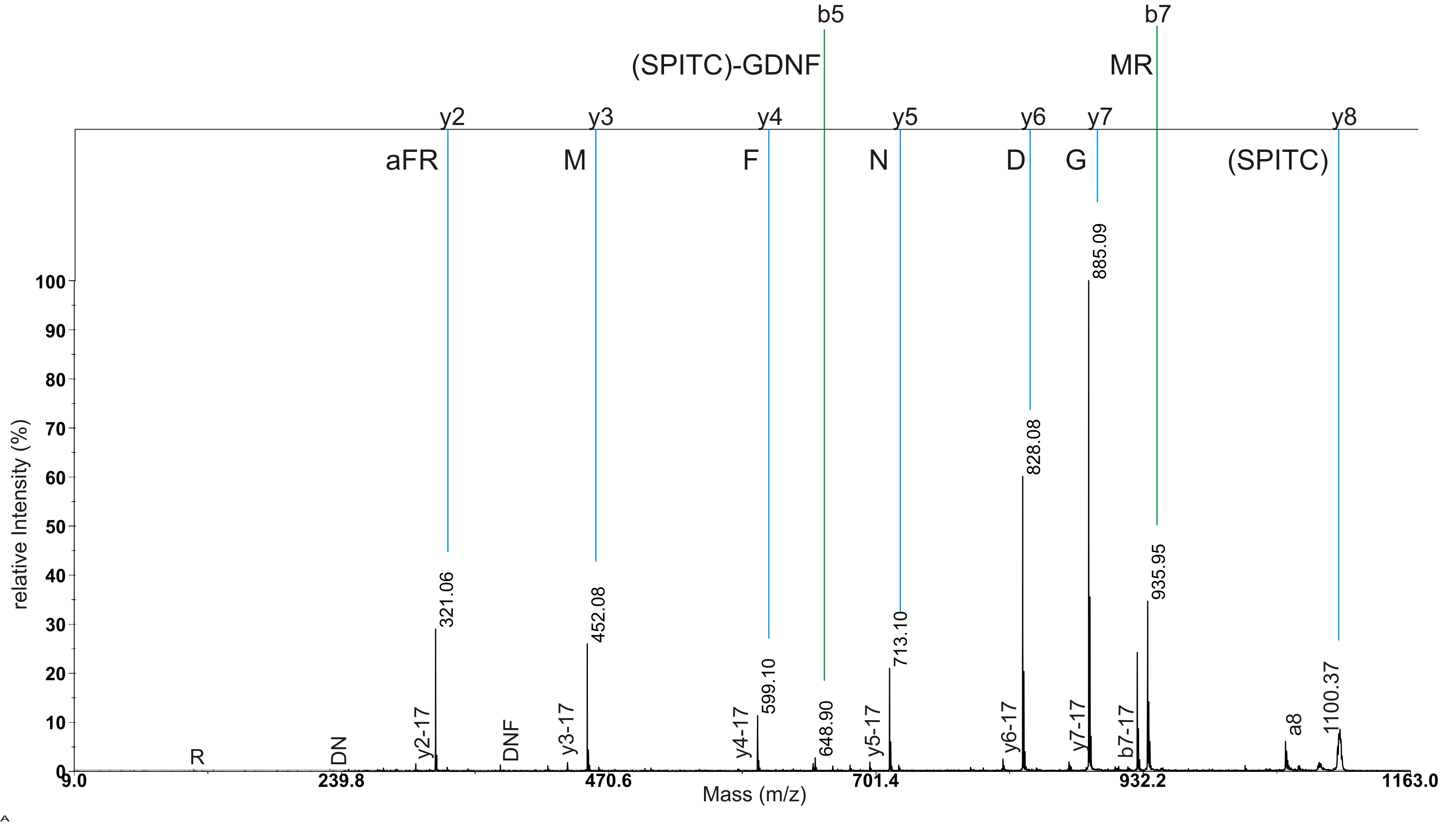

Supplement: Figure S3 — MS/MS spectrum of FMRFa885, SPITC-labelled. (TIF) [file pone.0041543.s003.tif]

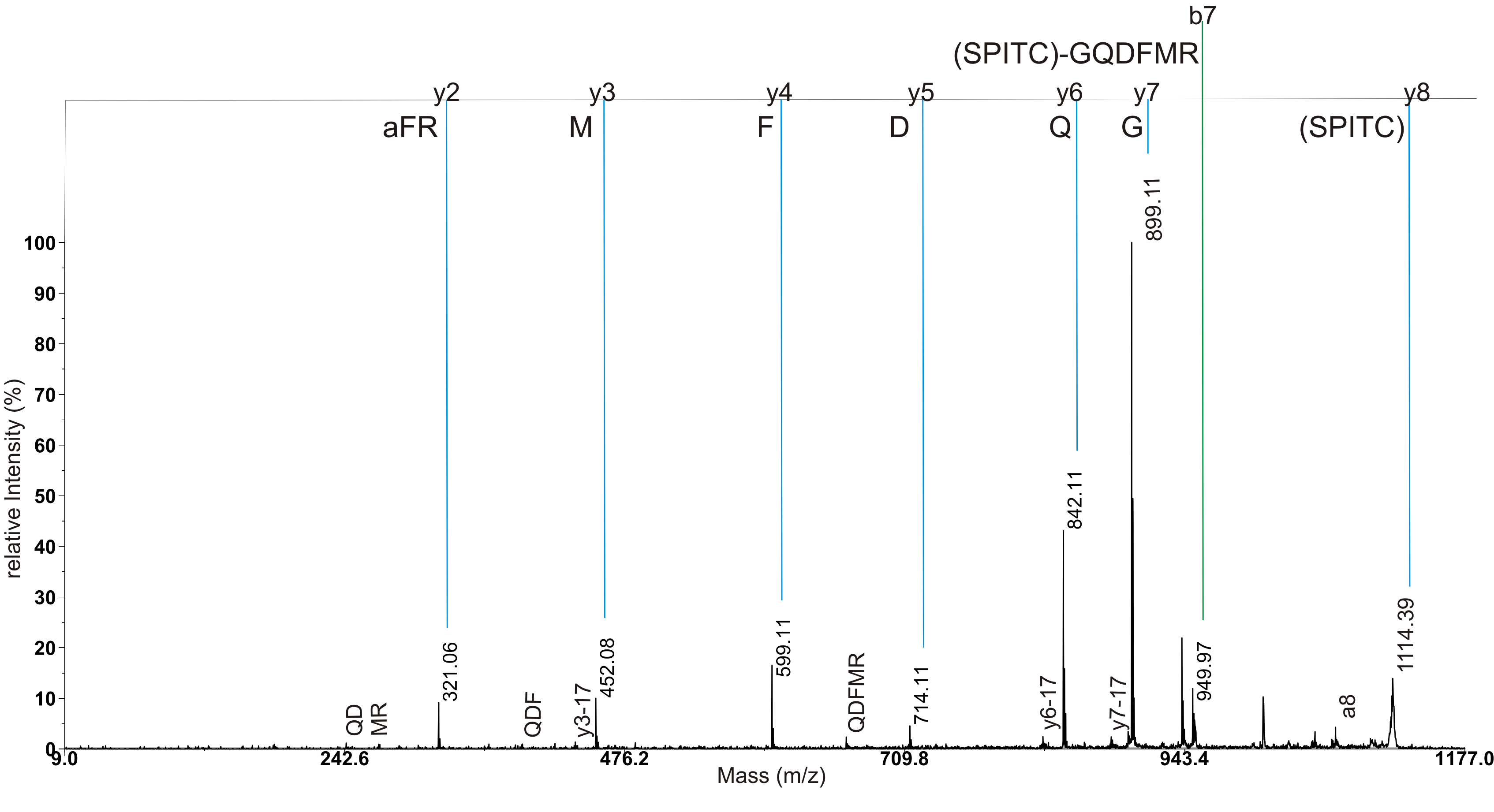

Supplement: Figure S4 — MS/MS spectrum of FMRFa899, SPITC-labelled. (TIF) [file pone.0041543.s004.tif]

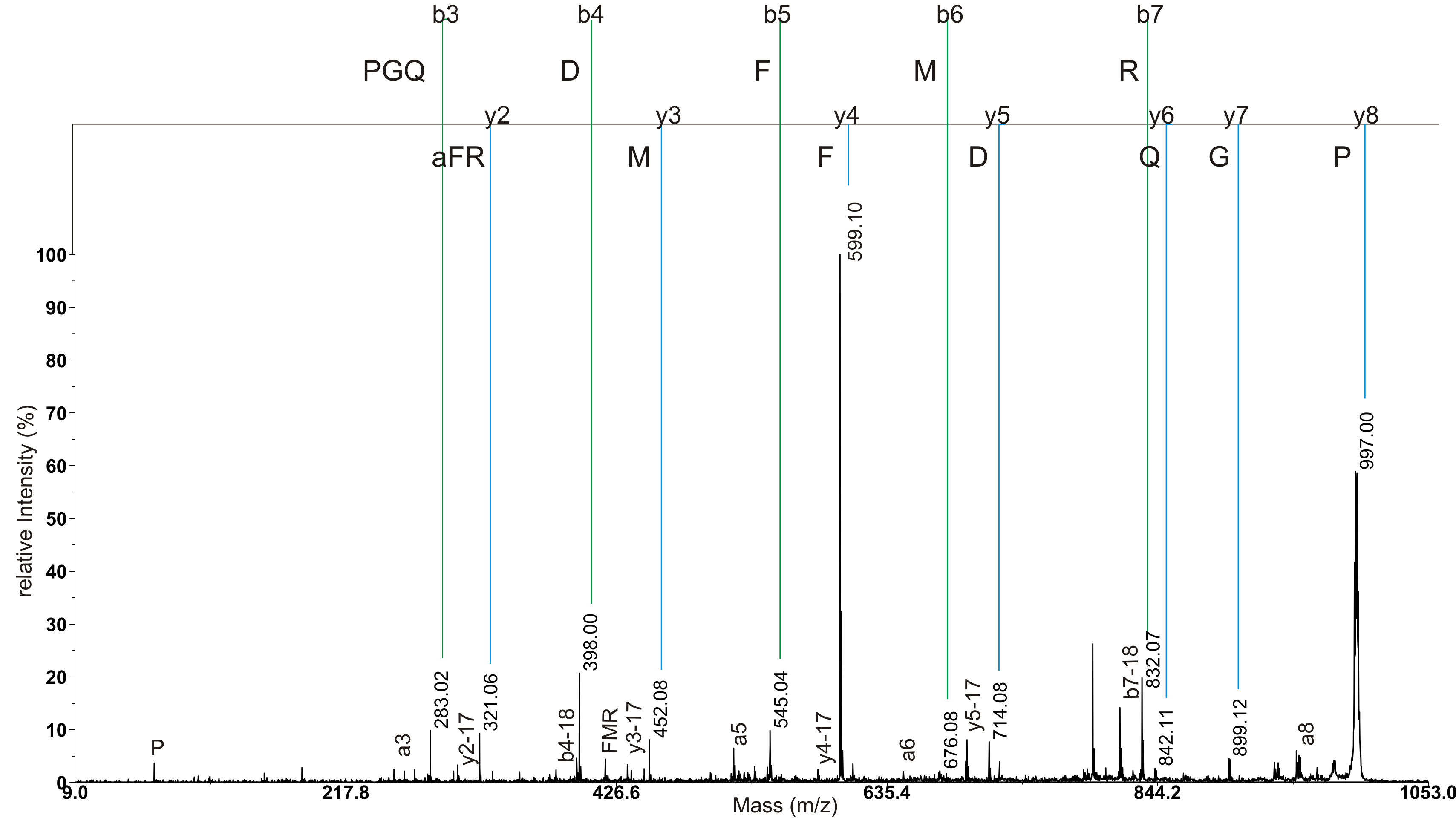

Supplement: Figure S5 — MS/MS spectrum of FMRFa996, unlabeled. (TIF) [file pone.0041543.s005.tif]

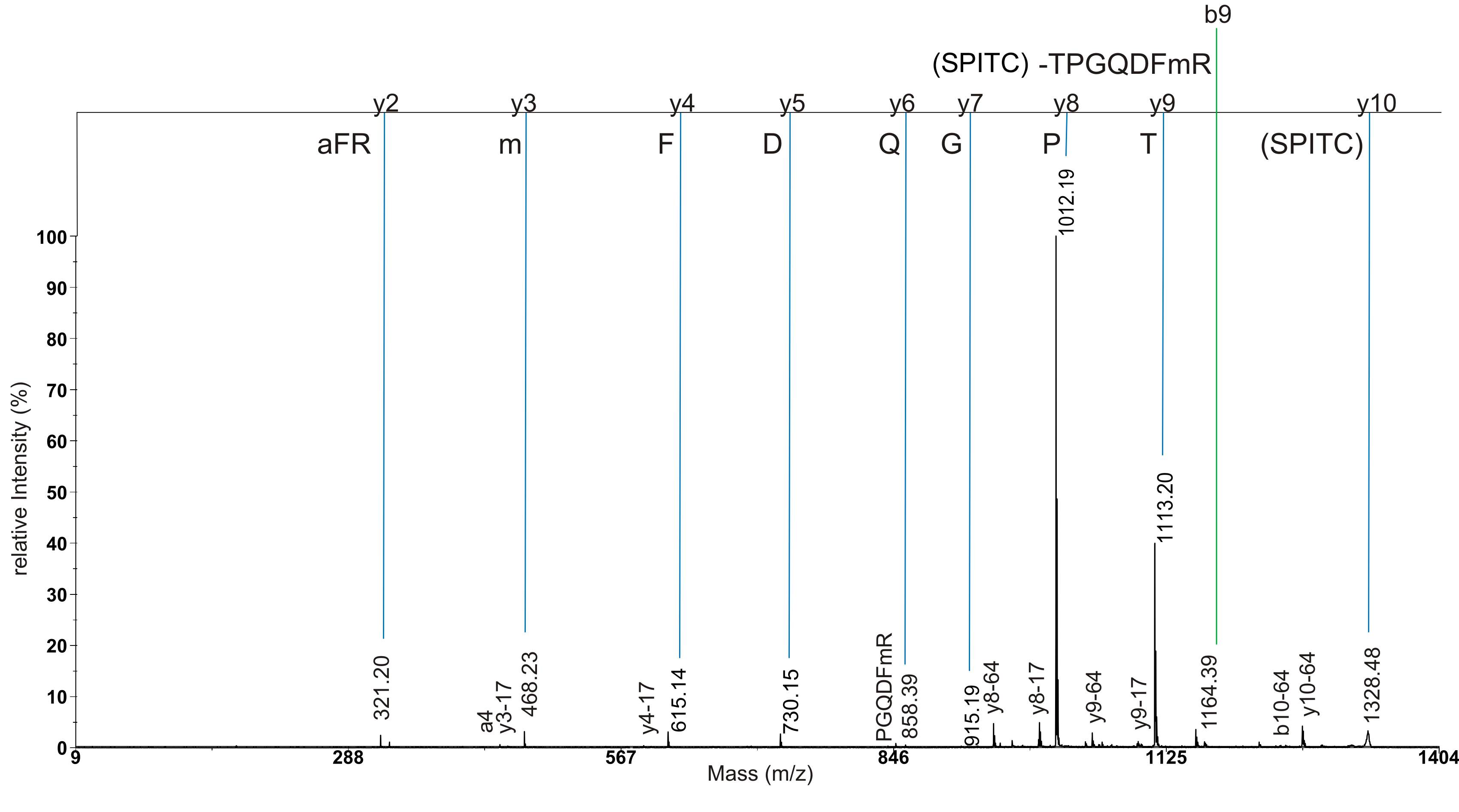

Supplement: Figure S6 — MS/MS spectrum of FMRFa1097 with an oxidised methionine (1113.5 Da), SPITC-labelled. (TIF) [file pone.0041543.s006.tif]

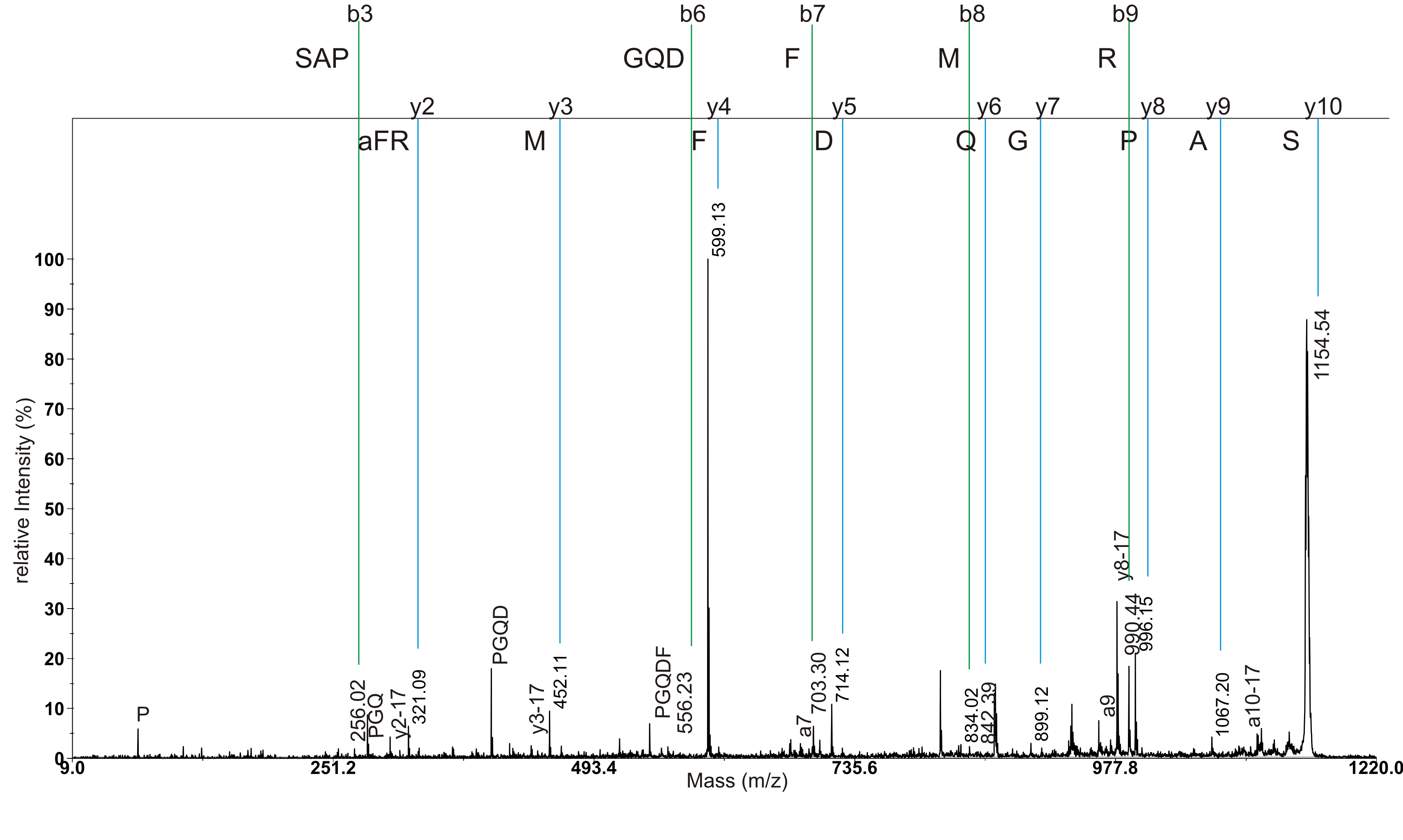

Supplement: Figure S7 — MS/MS spectrum of FMRFa1154, unlabeled. (TIF) [file pone.0041543.s007.tif]

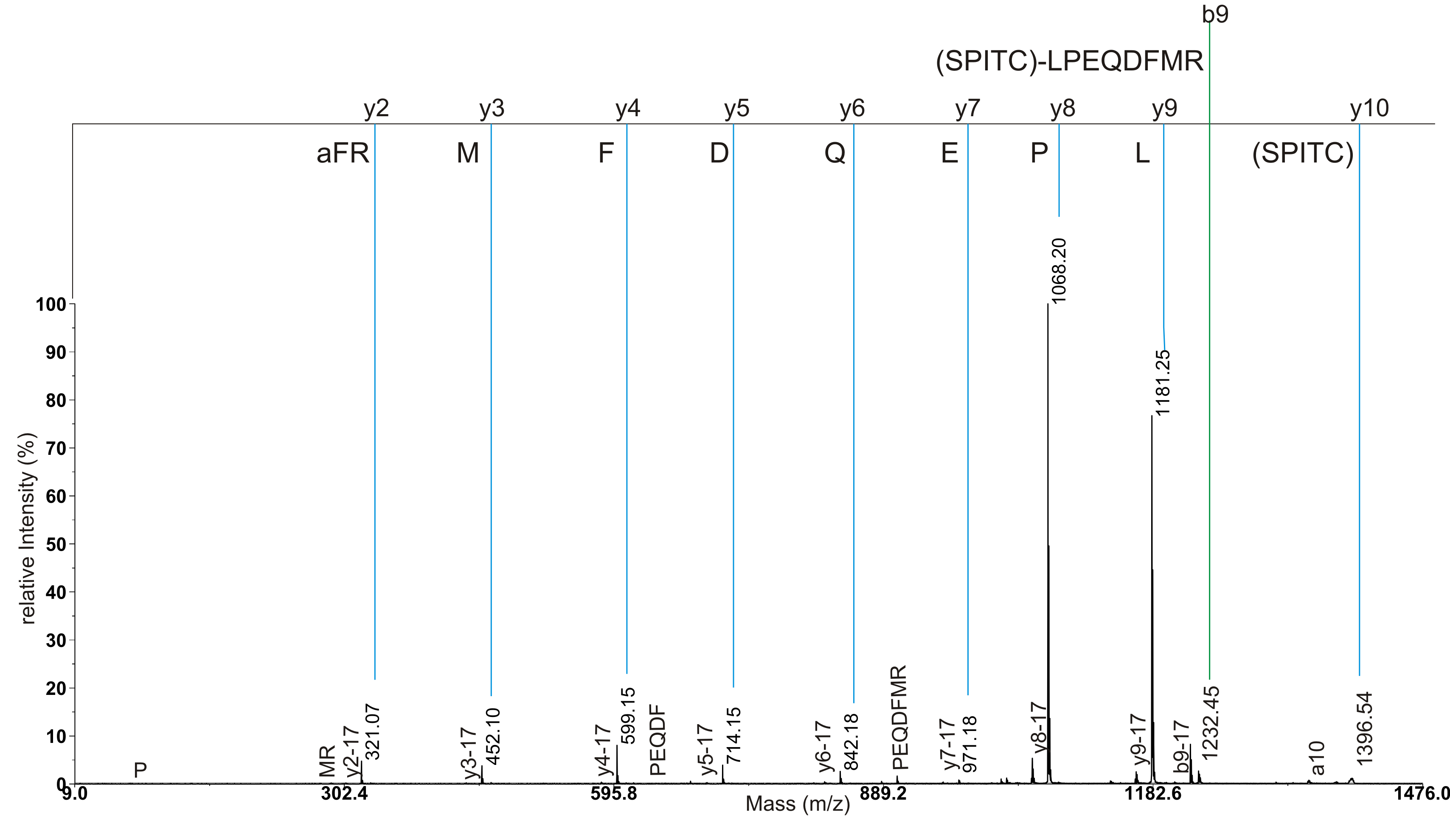

Supplement: Figure S8 — MS/MS spectrum of FMRFa1181, SPITC-labelled. (TIF) [file pone.0041543.s008.tif]

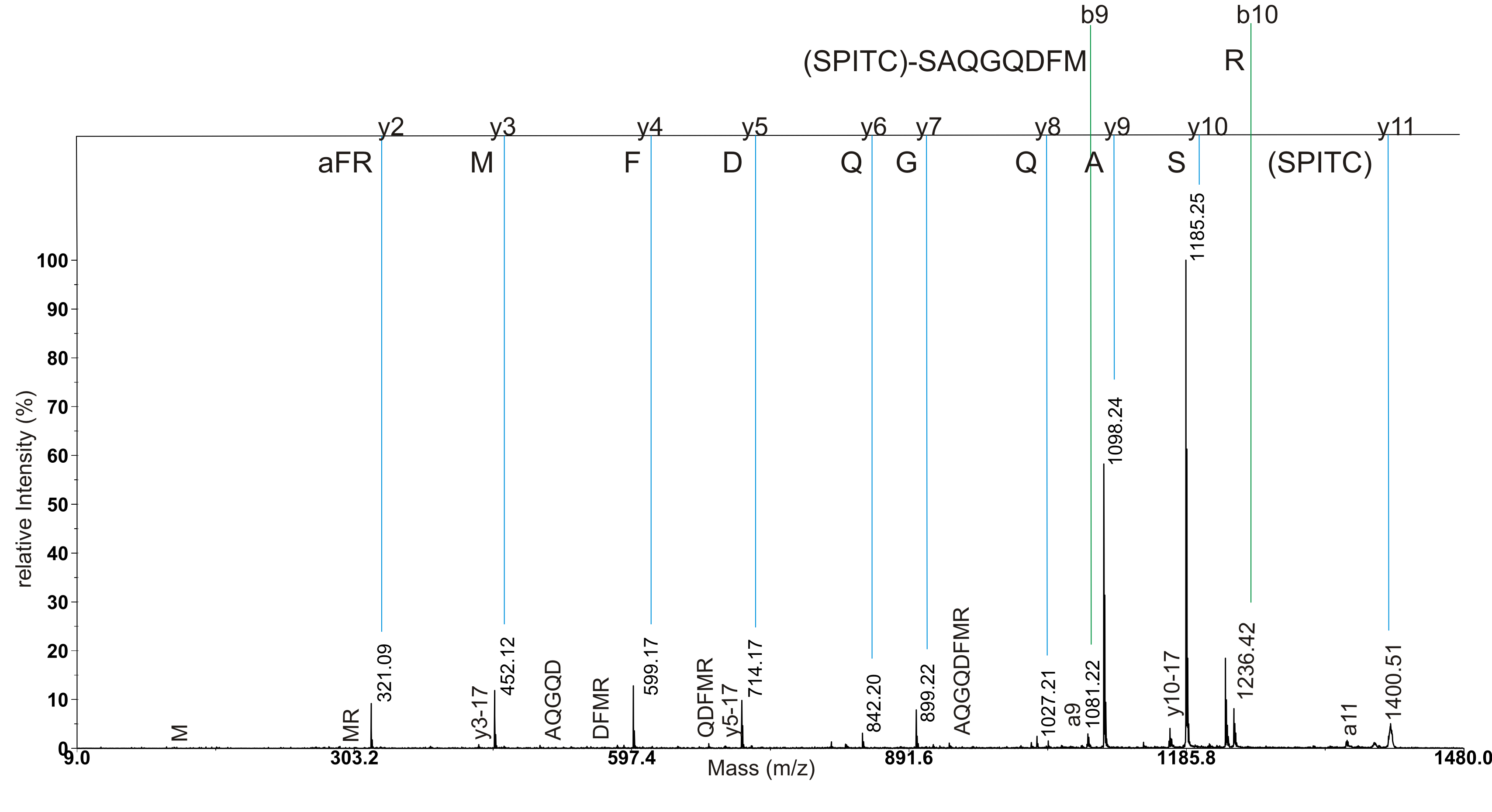

Supplement: Figure S9 — MS/MS spectrum of FMRFa1185, SPITC-labelled. (TIF) [file pone.0041543.s009.tif]

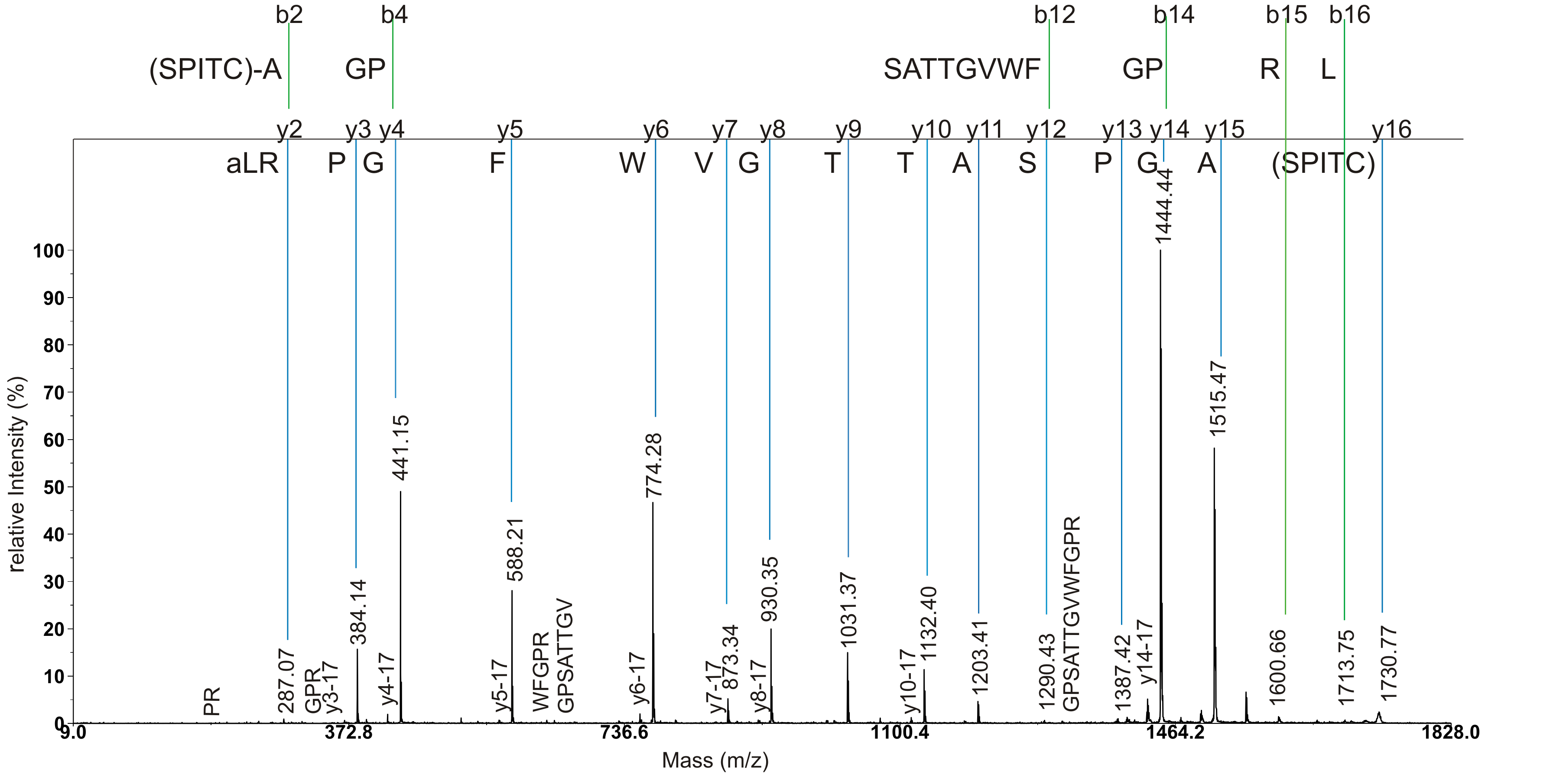

Supplement: Figure S10 — MS/MS spectrum of CAPA-PK, SPITC-labelled. (TIF) [file pone.0041543.s010.tif]

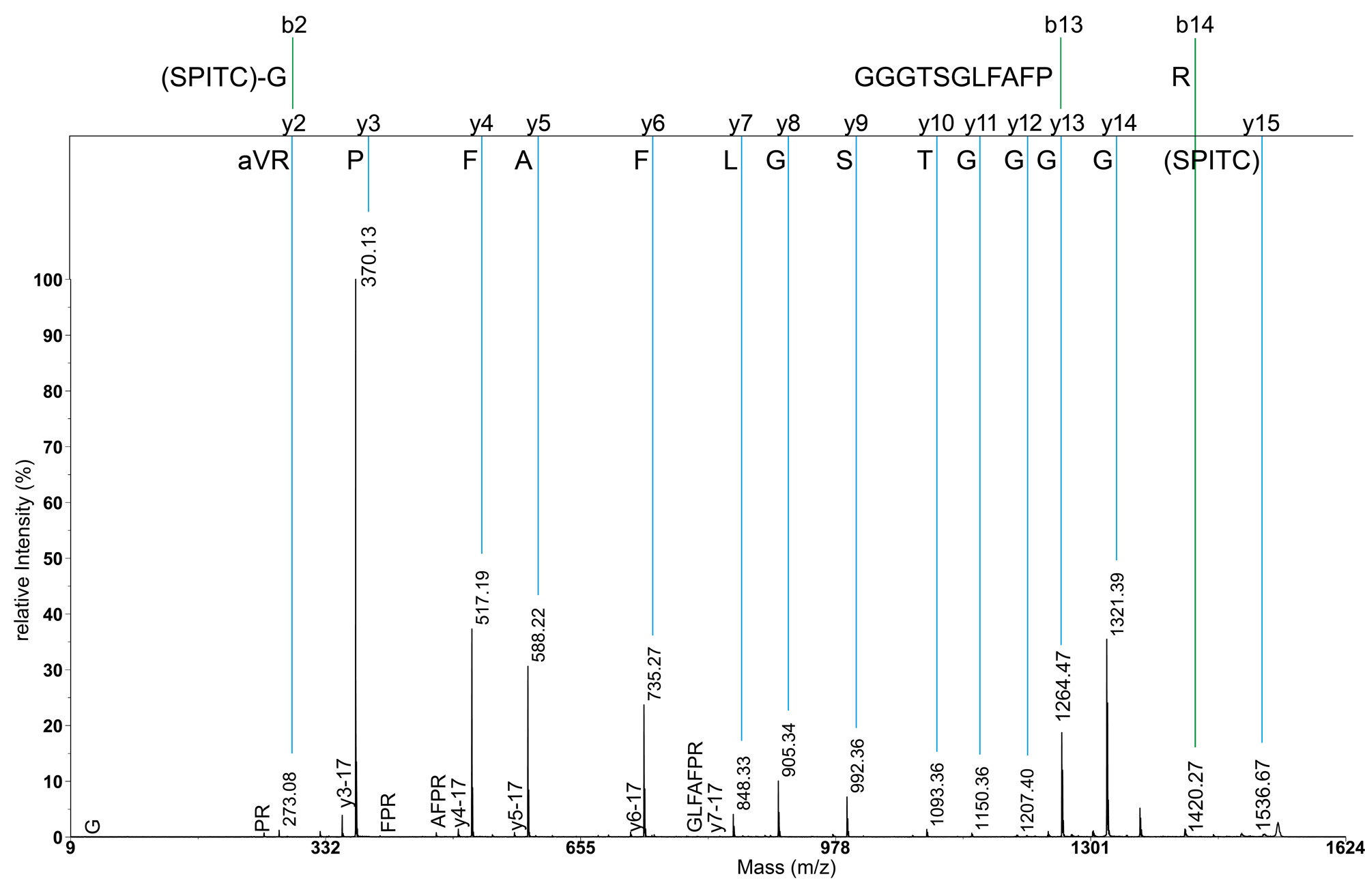

Supplement: Figure S11 — MS/MS spectrum of CAPA-PVK-1, SPITC-labelled. (TIF) [file pone.0041543.s011.tif]

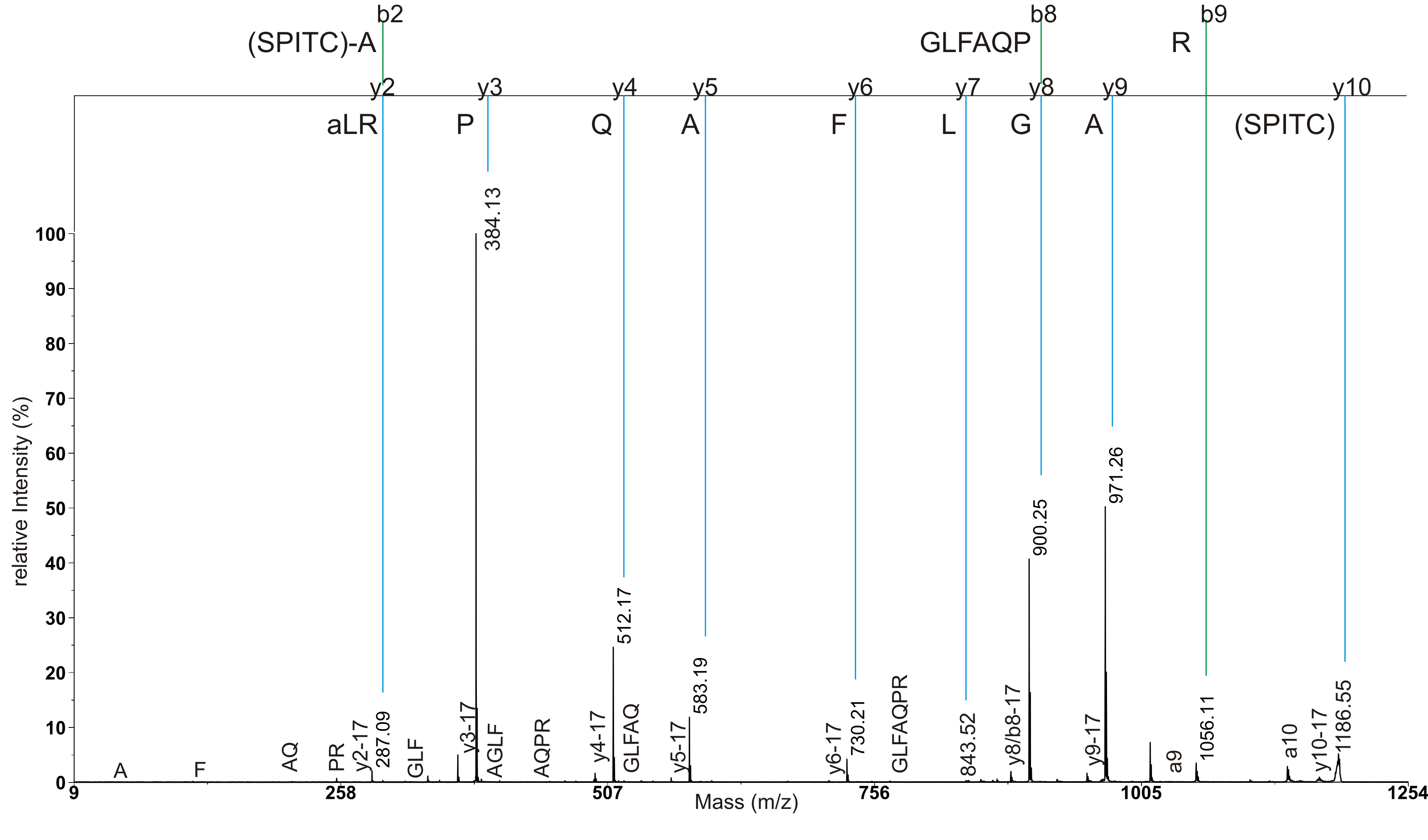

Supplement: Figure S12 — MS/MS spectrum of CAPA-PVK-2, SPITC-labelled. (TIF) [file pone.0041543.s012.tif]

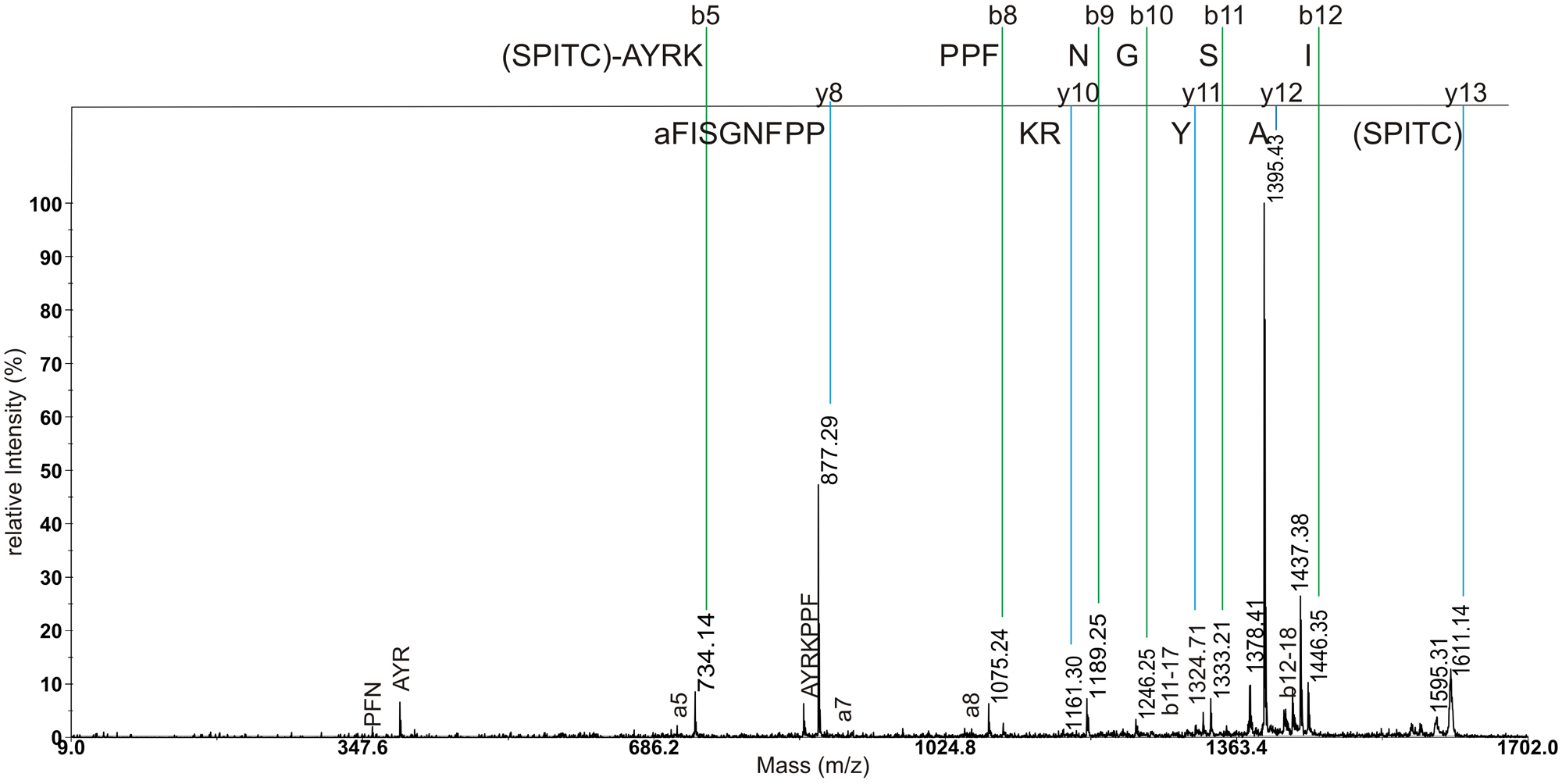

Supplement: Figure S13 — MS/MS spectrum of SIFa, SPITC-labelled. (TIF) [file pone.0041543.s013.tif]

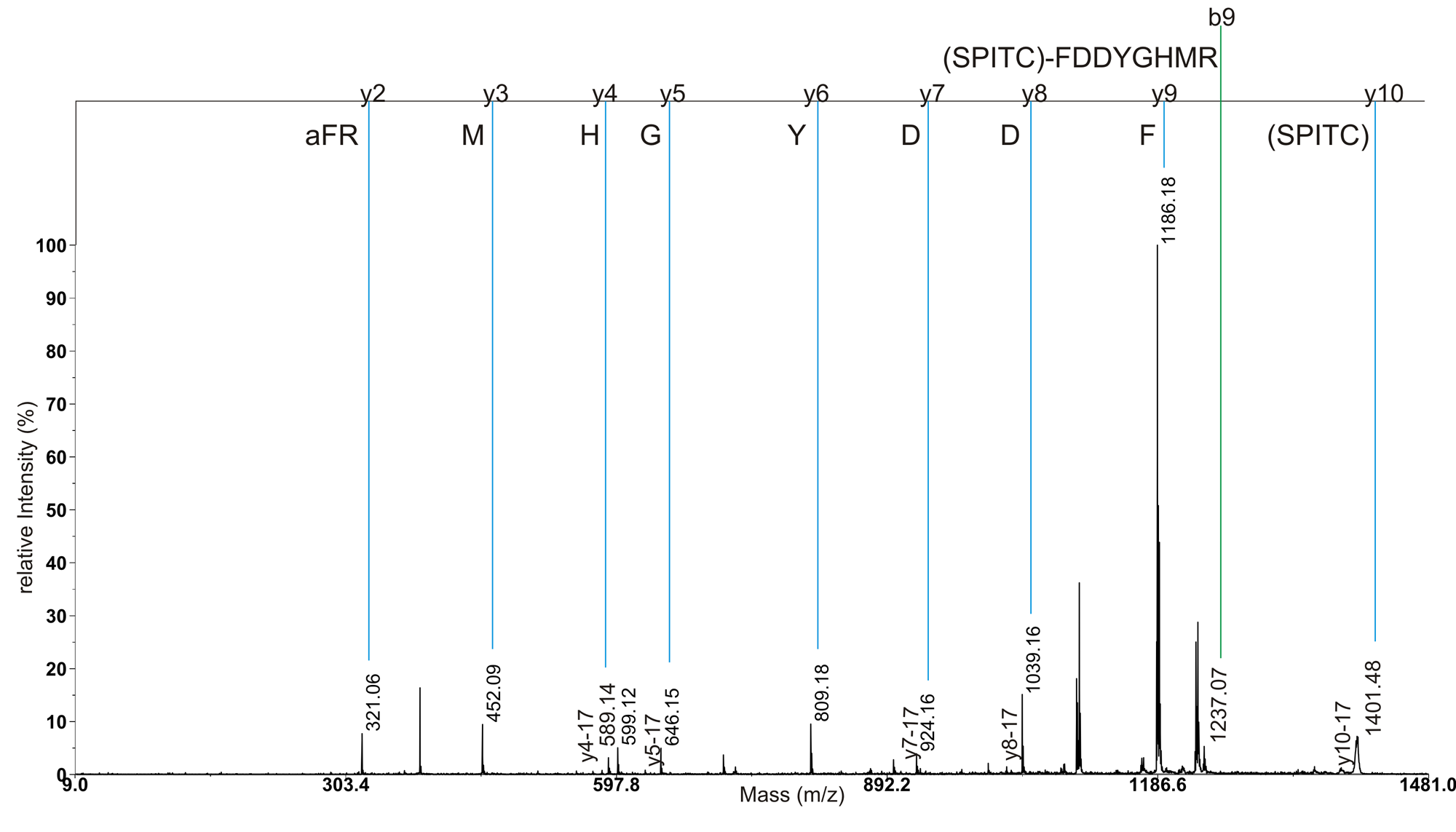

Supplement: Figure S14 — MS/MS spectrum of sulfakinin6-14, SPITC-labelled. (TIF) [file pone.0041543.s014.tif]

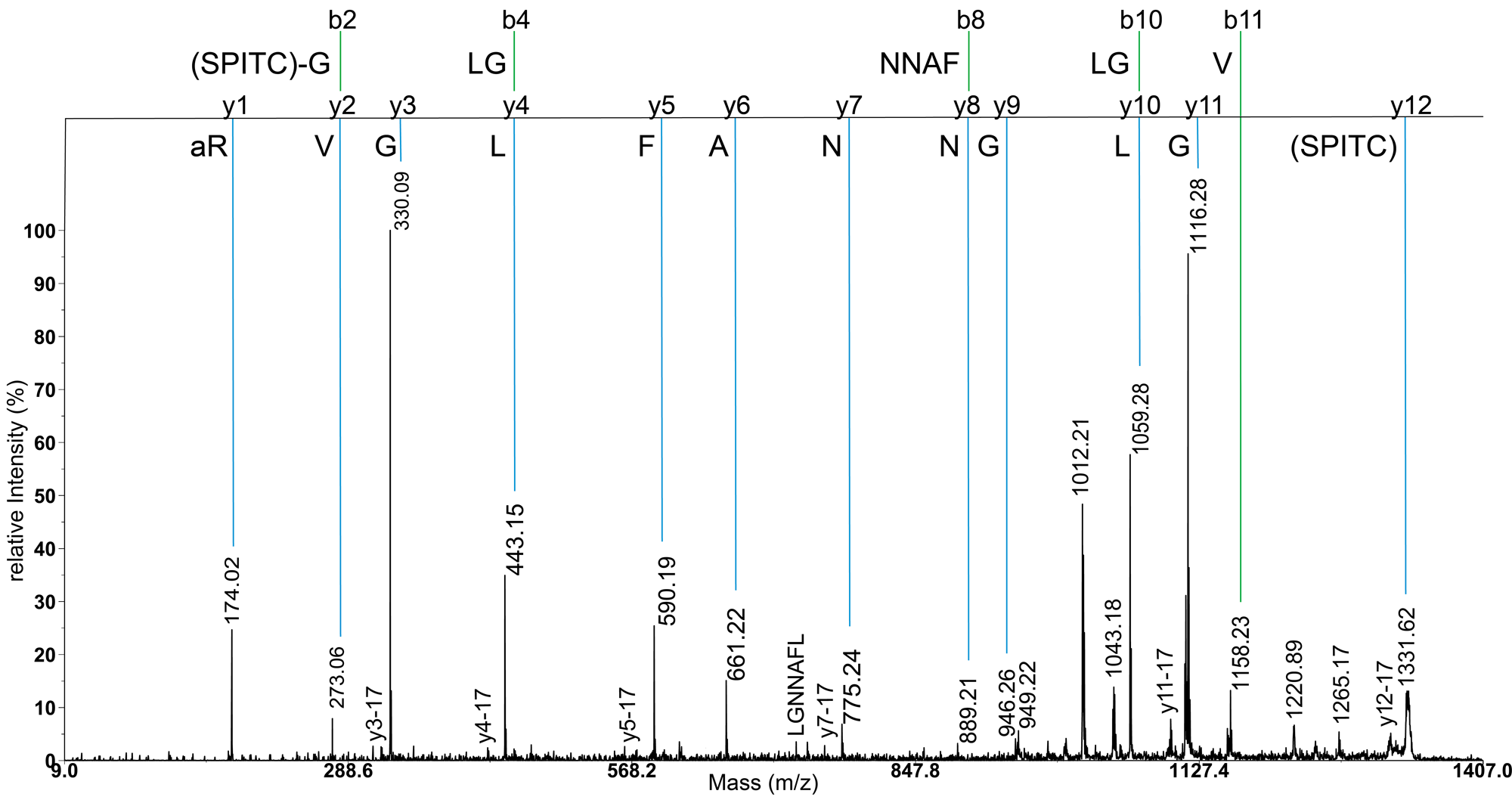

Supplement: Figure S15 — MS/MS spectrum of TK1116, SPITC-labelled. (TIF) [file pone.0041543.s015.tif]

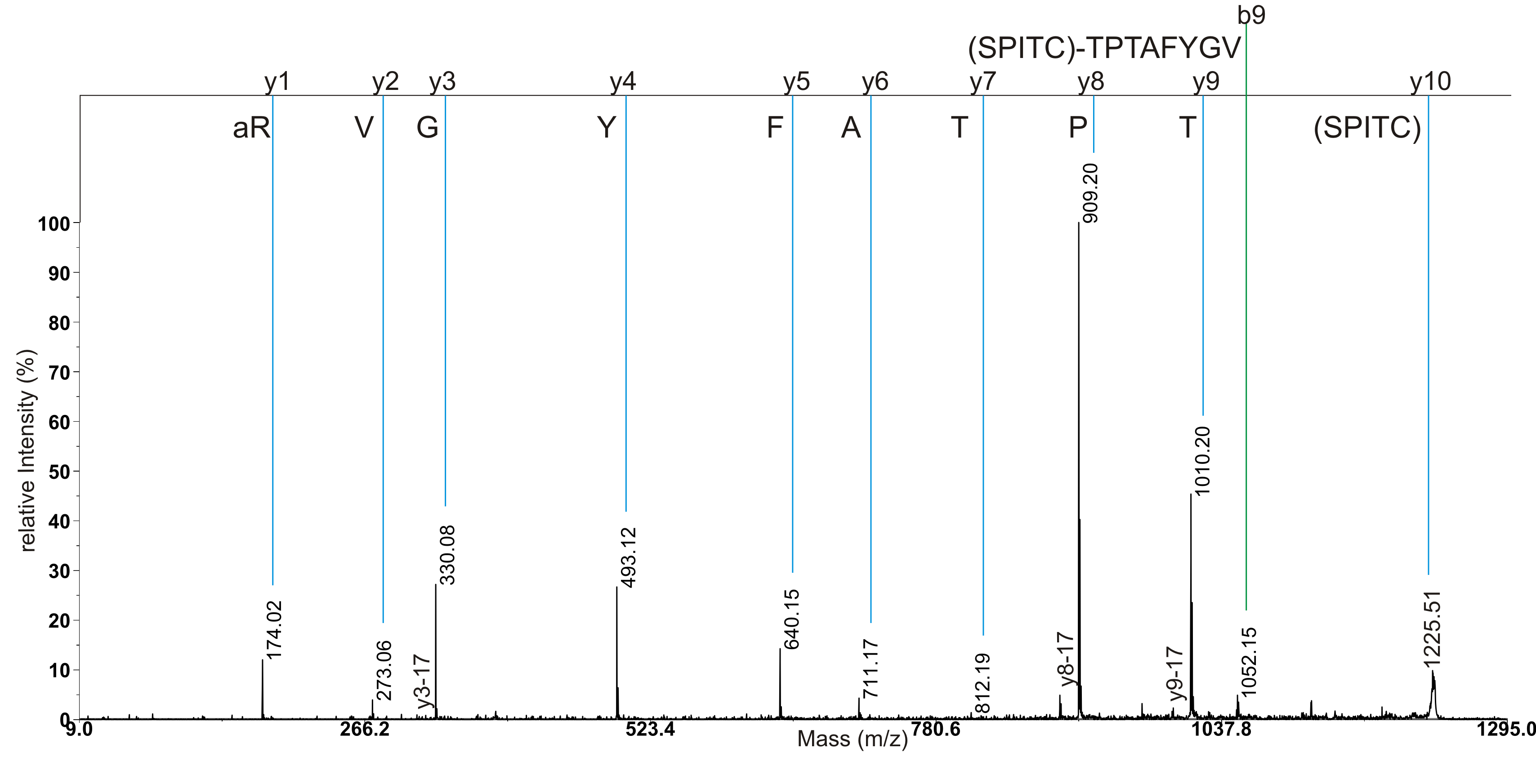

Supplement: Figure S16 — MS/MS spectrum of TK1010, SPITC-labelled. (TIF) [file pone.0041543.s016.tif]

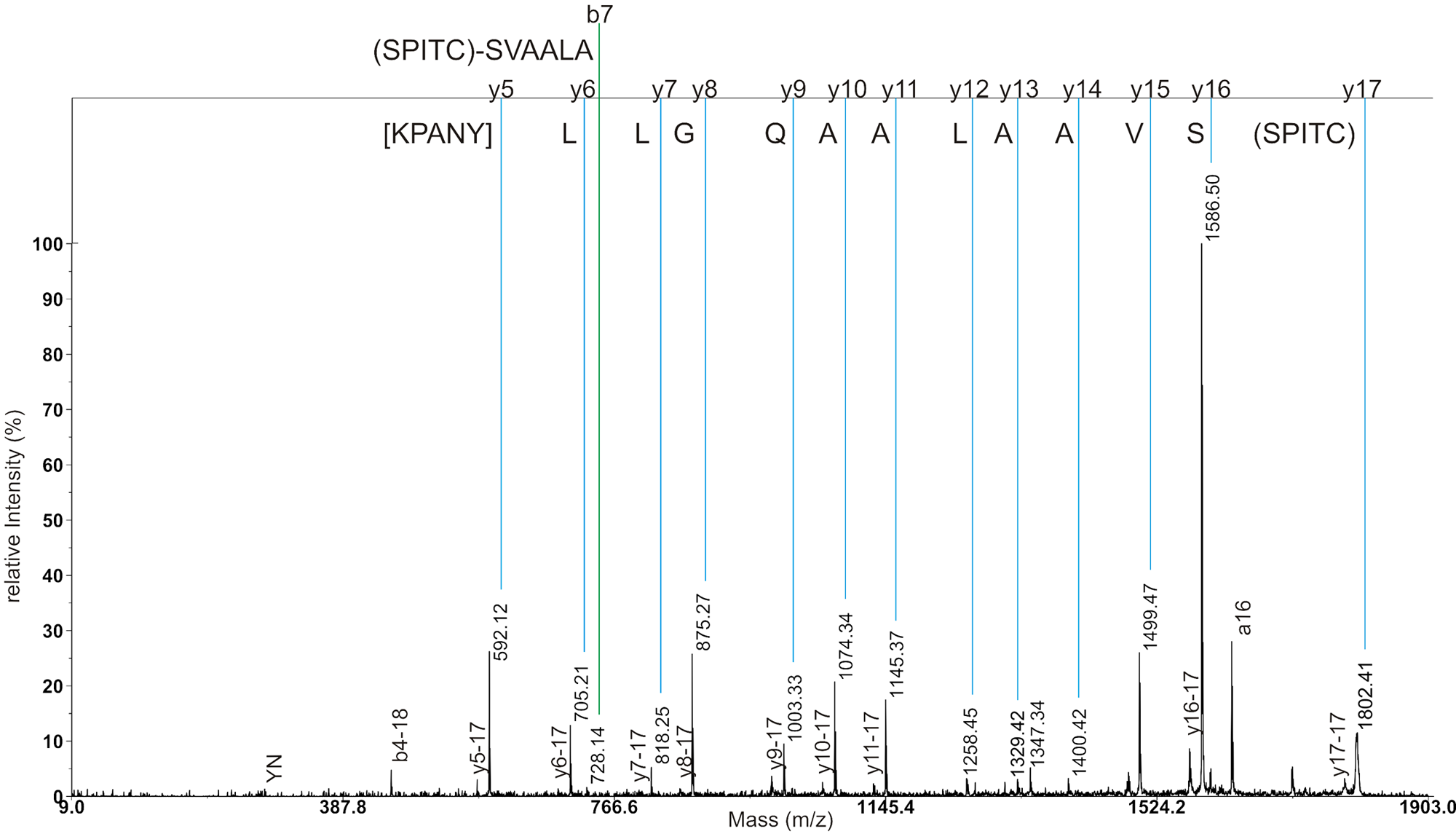

Supplement: Figure S17 — MS/MS spectrum of APK, SPITC-labelled. (TIF) [file pone.0041543.s017.tif]
